# The diagnostic validity of musculoskeletal ultrasound in lateral epicondylalgia: a systematic review

**DOI:** 10.1186/1471-2342-14-10

**Published:** 2014-03-03

**Authors:** Valentin C Dones, Karen Grimmer, Kerry Thoirs, Consuelo G Suarez, Julie Luker

**Affiliations:** 1 International Centre for Allied Health Evidence, University of South Australia, Adelaide, South Australia; 2 Division of Health Sciences, University of South Australia, C8-26, Centenary Building, GPO Box 2471, Adelaide, SA 5001, Australia; 3 Department of Rehabilitation Medicine, Faculty of Medicine and Surgery, University of Santo Tomas, Manila, Philippines

**Keywords:** Systematic review, Diagnosis, Musculoskeletal ultrasound, Lateral epicondylalgia, Lateral epicondylitis, Tennis elbow

## Abstract

**Background:**

Ultrasound is considered a reliable, widely available, non-invasive and inexpensive imaging technique for assessing soft tissue involvement in Lateral epicondylalgia. Despite the number of diagnostic studies for Lateral Epicondylalgia, there is no consensus in the current literature on the best abnormal ultrasound findings that confirm lateral epicondylalgia.

**Methods:**

Eligible studies identified by searching electronic databases, scanning reference lists of articles and chapters on ultrasound in reference books, and consultation of experts in sonography. Three reviewers (VCDIII, KP, KW) independently searched the databases using the agreed search strategy, and independently conducted all stages of article selection. Two reviewers (VCDIII, KP) then screened titles and abstracts to remove obvious irrelevance. Potentially relevant full text publications which met the inclusion criteria were reviewed by the primary investigator (VCDIII) and another reviewer (CGS).

**Results:**

Among the 15 included diagnostic studies in this review, seven were Level II diagnostic accuracy studies for chronic lateral epicondylalgia based on the National Health and Medical Research Council Hierarchy of Evidence. Based from the pooled sensitivity of abnormal ultrasound findings with homogenous results (p > 0.05), the hypoechogenicity of the common extensor origin has the best combination of diagnostic sensitivity and specificity. It is moderately sensitive [Sensitivity: 0.64 (0.56-0.72)] and highly specific [Specificity: 0.82 (0.72-0.90)] in determining elbows with lateral epicondylalgia. Additionally, bone changes on the lateral epicondyle [Sensitivity: 0.56 (0.50-0.62)] were moderately sensitive to chronic LE. Conversely, neovascularity [Specificity: 1.00 (0.97-1.00)], calcifications [Specificity: 0.97 (0.94-0.99)] and cortical irregularities [Specificity: 0.96 (0.88-0.99)] have strong specificity for chronic lateral epicondylalgia. There is insufficient evidence supporting the use of Power Doppler Ultrasonogrophy, Real-time Sonoelastography and sonographic probe-induced tenderness in diagnosing LE.

**Conclusions:**

The use of Gray-scale Ultrasonography is recommended in objectively diagnosing lateral epicondylalgia. The presence of hypoechogenicity and bone changes indicates presence of a stressed common extensor origin-lateral epicondyle complex in elbows with lateral epicondylalgia. In addition to diagnosis, detection of these abnormal ultrasound findings allows localization of pathologies to tendon or bone that would assist in designing an appropriate treatment suited to patient’s condition.

## Background

Lateral Epicondylalgia is the most common cause of lateral elbow pain [1]. It is generally attributed to osteotendinous irritation of the common extensor origin in which pathological changes in the tendinous origins of Extensor Carpi Radialis Brevis (ECRB) and Extensor Digitorum Communis (EDC) muscles [2-8] are commonly implicated.

In this review, the term ‘Lateral epicondylalgia’ represents pain in the lateral elbow area involving the lateral epicondyle and the common extensor origin regardless of the nature (inflammatory or non-inflammatory), acuity of elbow symptoms (acute or chronic) and other sources of pathology. Considering the term ‘Lateral epicondylalgia’ is irrelevant to the nature, acuity and sources of pathology of lateral elbow symptoms [9], the name ‘Lateral epicondylalgia’ encompasses lateral epicondylitis [10], lateral elbow tendinosis [11], lateral elbow enthesopathy [12,13], or lateral elbow epicondylopathy [14].

There is currently no gold standard in diagnosing Lateral epicondylalgia [15]. However in both clinical practice and research, the Cozen, Mill, and Maudsley tests are commonly used provocation tests which are considered positive if they replicate lateral elbow pain [15]. The Cozen test is the only one recommended by the United Kingdom Health Safety Executive Workshop to diagnose Lateral epicondylalgia [16]. However, the diagnostic capacity of these three clinical provocation tests to confirm Lateral epicondylalgia is under-investigated [15].

Consequently, health care professionals are increasingly using musculoskeletal ultrasound to identify tendon pathologies which may be associated with Lateral Epicondylalgia [17]. Musculoskeletal Ultrasound is reported to be reliable, widely available, non-invasive and inexpensive [17].

Gray-scale Ultrasonography is the most commonly used musculoskeletal ultrasound in detecting pathological changes in tendons [17]. It was suggested by Grassi et al. to be the reference standard for diagnostic imaging in rheumatologic conditions [17]. However, there is currently no consensus on the best musculoskeletal ultrasound finding to confirm Lateral epicondylalgia.

In Gray-scale Ultrasonography, a high resolution and high frequency transducer is essential to clearly demonstrate the special resolution of superficial soft tissue structures [18]. The echotexture of muscles, tendon and bones have been observed differently in real-time, using transducer heads with varying frequency bands, for instance:

● Gibbon: 7.5 or 10 MHz [18]

● Bianchi and Martinoli: 5–15 MHz [19] and

● Robinson: 9–17 MHz [20]

Another musculoskeletal ultrasound technology has recently emerged to objectively assess Lateral epicondylalgia, namely Power Doppler Ultrasonography and Real-time Sonoelastography. The poor scanning ability of the Colour Doppler Ultrasonography in detecting slow blood flow and separating blood flow from background noise is addressed by the Power Doppler Ultrasonography. Power Doppler Ultrasonography is useful when optimal Doppler angles (of 60 degrees or less) cannot be obtained. It scans longer segments of vessels and more individual vessels [21]. With its improved ability to detect increased blood flow, it appears to have the highest diagnostic validity for chronic Lateral epicondylalgia [22,23]. Real-Time Sonoelastography assesses tissue elasticity through compression [22]. With the common extensor origin suspected of weakening due to intratendinous focal changes, its compressibility is increased compared to healthy tendons indicating tendon degeneration [22]. De Zordo et al. [22] and Khoury and Cardinal [23] suggested Real-time Sonoelastography can be a powerful adjunct to the diagnosis of Lateral epicondylalgia.

A manual technique which evolved from the use of musculoskeletal ultrasound is sonographic probe-induced tenderness. During musculoskeletal ultrasound scan, the operator uses a small part of one end of the musculoskeletal ultrasound probe which is equivalent to the tip of the index finger on a painful elbow specifically where a musculoskeletal ultrasound pathologic lesion (i.e. hypoechogenicity) is reported. The musculoskeletal ultrasound lesion is found relevant only if tenderness is elicited [24]. This intervention was suggested to increase the accuracy of identifying the exact zone of abnormality and useful in confirming the location of the pathology in elbows with Lateral epicondylalgia [24].

### Aims

This review was primarily undertaken with the aims of establishing the diagnostic validity of Gray-scale Ultrasonography (the index test) for Lateral epicondylalgia, using a clinical (provocation test) diagnosis of Lateral epicondylalgia as the reference standard. A secondary aim was to establish any improvements in diagnostic sensitivity of musculoskeletal ultrasound in determining Lateral epicondylalgia when using Colour Doppler Ultrasonography, Power Doppler Ultrasonography, Real-time Sonoelastography, sonographic probe-induced tenderness or high frequency transducer head.

## Methods

### Eligibility

Studies were included if they reported on humans with Lateral epicondylalgia, reported at least one clinical provocation testing as a reference standard, and reported any statistic relating to the diagnostic validity of musculoskeletal ultrasound for Lateral epicondylalgia. Diagnostic validity could be available using any estimate (e.g. sensitivity, specificity, likelihood ratio or predictive values) or if these could be calculated from available data. There were no age restrictions on participants.

Literature was searched between January 1990 and May 2013, this time period reflecting the evolution of musculoskeletal ultrasound equipment and techniques. Studies that included participants diagnosed with other types of lateral elbow pain such as fibromyalgia or osteoarthritis were excluded.

### Information sources

Eligible studies were identified by primary and secondary searching. Primary searching involved a comprehensive search of EMBASE, OVID, ICONDA, International Pharmaceutical Abstracts, Cochrane and DARE (Database of Abstracts of Reviews of Effectiveness), PUBMED, Google Scholar, Web of Science, Web of Knowledge, EBSCO (CINAHL, SPORTDiscus, Academic Search Premier, Health Source: Nursing/Academic Edition, ERIC, PsycInfo), Science Direct databases, HighWire Press, PubMed Central, Scopus, PsycARTICLES, Informit e-library collections, Biomed Central Gateway, and TRIP Database were searched. No language limitations were applied. Secondary searching involved pearling reference lists of published articles, or chapters on ultrasound in reference books, and by consulting experts in the field of sonography.

### Search strategy

Boolean terms and three sets of keywords were used in search strategies which included:

Keywords 1: sensitivity OR specificity OR diagnostic accuracy OR diagnosis OR accuracy, AND

Keywords 2: lateral epicondylitis OR tennis elbow OR radial epicondyalgia OR lateral epicondylalagia OR extensor tendinopathy OR epicondylitis lateralis humeris OR lateral elbow tendinopathy OR lateral epicondylosis OR tennis elbow OR lateral tennis elbow, AND

Keywords 3: sonography OR ultrasound OR musculoskeletal ultrasound

### Study selection

**Figure 1:**
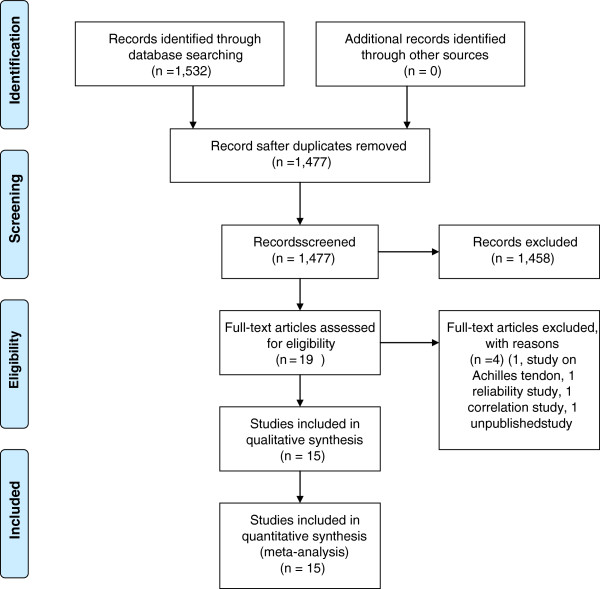
**The PRISMA flow diagram.***Key:* PRISMA, Preferred Reporting Items for Systematic Reviews and Meta-Analyses.

Three reviewers (VCDIII, KP, KW) independently searched the databases using the agreed search strategy, and independently conducted all stages of article selection. Two reviewers (VCDIII, KP) then screened titles and abstracts and agreed on 19 articles possibly relevant to this review. Full texts were retrieved. Studies were then reviewed by VCDIII and another reviewer (CGS).The PRISMA flow diagram illustrated the process of identifying relevant studies used in this systematic review (Figure 1). At all stages of the review process, the reviewers reached consensus by discussion. A third independent reviewer was available for arbitration, but was not used.

### Hierarchy and methodological quality

The National Health and Medical Research Council (NHMRC) hierarchy for evidence for diagnostic studies was applied [25]. As recommended by Fontela et al. [26], the Quality Assessment for Diagnostic Accuracy Studies (QUADAS) [27] (Additional file 1: Appendix 1. QUADAS checklist) and the Standards for Reporting of Diagnostic Accuracy (STARD) [28] (Additional file 1: Appendix 2. STARD checklist) and tools were applied to appraise the methodological quality. The STARD identified the quality of reporting study procedures and results, and QUADAS graded the methodological quality. To improve the rigor in grading STARD, relevant data were extracted prior to grading an item (Additional file 1: Appendix 2. STARD checklist). Each item was graded as well covered, adequately addressed, poorly addressed, and not addressed. Items which were assigned scores of well covered and adequately addressed were given a score of 1, otherwise 0.

The full text included articles were independently appraised by two reviewers; QUADAS [27] (VCDIII, JL) and STARD [28] (VCDIII, CGS). All disagreements were resolved during discussion without intervention from a third party.

### Data extraction process

Data extraction was performed independently by VCDIII and CGS using a specifically-designed data extraction tool, which integrated STARD (Additional file 1: Appendix 2. STARD checklist). Data were extracted on authors, year, country where study was performed, characteristics of the study population, sample size, inclusion and exclusion criteria, details of index texts used and results. Missing data were requested through e-mail from corresponding authors, by the primary author. Only authors of one paper provided missing data.

### Data analysis

Where statistical estimates of diagnostic validity were missing, raw data were extracted from frequency tables by the primary author, or were derived from published sensitivity and specificity results Calculations were verified by an epidemiologist (KG).

### Pooling of musculoskeletal ultrasound findings

Data were pooled where possible, to determine the standardised sensitivity and specificity of musculoskeletal ultrasound administrations (Gray-scale Ultrasonography, Colour Doppler Ultrasonography, Power Doppler Ultrasonography and Real-time Sonoelastography). To appropriately pool the musculoskeletal ultrasound results, two steps were taken to identify similar studies. The first step identified the studies which used the same criteria in determining a musculoskeletal ultrasound abnormality. From this subset of studies, the second step identified those which used similar descriptions of: a. inclusion criteria, b. type and frequency of transducer head used, c. qualification of the reader of the image, d. duration of elbow symptoms, e. age of the participant, and f. the reference standard used.

MetaDisc 1.4 was used to compute for the pooled sensitivity and specificity and heterogeneity of musculoskeletal ultrasound findings from the subset of similar studies, described above [29]. P-values <0.05 indicated presence of significant heterogeneity in pooled results on sensitivity and specificity. Inconsistency square test for homogeneity (I^2^) (in percentage) was computed to determine the degree of variability in study results. It describes the percentage of variation across studies that are due to heterogeneity rather than chance [30]. I^2^ = 100% X (Q-df)/Q where Q is distributed as a chi-square statistic with K (number of studies) minus 1 degree of freedom and df is degrees of freedom [30].

### High frequency versus low frequency range

With the frequency range recommended by Bianchi and Martinoli [19] and Robinson [20], this review categorised the musculoskeletal ultrasound frequency into high frequency range of 9–17 MHz [20] and low frequency range of 5–15 MHz [19] and tested its ability to detect abnormal musculoskeletal ultrasound findings. Musculoskeletal ultrasound results of diagnostic studies were excluded if:

● The frequency of the transducer head used to scan the elbows was not mentioned in the study.

● The study used sonographic probe-induced tenderness to confirm the presence of musculoskeletal ultrasound findings in elbows with Lateral epicondylalgia. This maneuver replicates the elbow pain which could potentially influence the testers’ objectivity in localizing the pathology within the elbow and interpreting the obtained images.

The transducer head (high frequency or low frequency) that effectively detected presence of abnormal musculoskeletal ultrasound findings in elbows with Lateral epicondylalgia was recommended as the frequency range for diagnosing Lateral epicondylalgia.

## Results

### Study selection

The number of database ‘hits’ was comparable between the three independent researchers, indicating that it was unlikely that important studies had been missed. Titles and abstracts of potentially relevant articles from these ‘hits’ were similarly identified between the three independent searches. From a total of 1,532 potentially relevant citations from the database searches, 15 full-text studies were eligible for inclusion. The study consort diagram is outlined in Figure 1 and hits identified per database are provided in Additional file 1: Appendix 3. Search results.

### Levels of evidence

No systematic reviews on diagnostic validity of musculoskeletal ultrasound findings on Lateral epicondylalgia elbows were found. Seven studies were classified as level II, five studies as level III-1, and three studies as level III-2. Each level corresponded with the description of included studies and the articles identified were reported in Additional file 1: Appendix 4. NHMRC Hierarchy of Evidence.

### Agreement between assessors

The agreement in STARD scores between the two reviewers (VCDIII, CGS) was good (weighted k = 0.671 (95% confidence interval 0.590-0.752). Disagreements were principally due to either reading errors, or differences in interpretation.

However when using QUADAS, the agreement between reviewers (VCDIII, JL) was only fair [weighted k = 0.381 (95% confidence interval 0.242-0.520). Disagreements were most common in criteria 1, 2, 9, and 12. Before reconsidering the scores, the reviewers agreed on the following: a. criterion 1: studies which recruited a group of healthy controls to compare against the participants with Lateral epicondylalgia were graded as “no”; b. criterion 2: studies which itemised the inclusion and exclusion criteria were graded as “yes”; c. criterion 9: studies which gave minimum details on clinical examination procedure were graded as “yes”; d. criterion 12: knowledge of the tester of the symptomatic side were graded as “no.”

Standards for Reporting of Diagnostic Accuracy (STARD) grades are reported for each included paper in Additional file 1: Appendix 5. STARD Grades. Ten STARD items were reported in fewer than 50% studies: identification as diagnostic study (27%), report on reference standard and its rationale (47%), recruitment period (47%), use of reliability tests (13%), time interval between the clinical examination and musculoskeletal ultrasound (20%), severity of symptoms (20%), adverse events (7%), diagnostic accuracy and 95% confidence interval (27%), subgroup analysis (20%) and estimates of reliability (13%).

Quality Assessment for Diagnostic Accuracy Studies (QUADAS) scores are presented in Additional file 1: Appendix 6. Quality Assessment for Diagnostic Accuracy Studies (QUADAS) scores. Forms of bias found in the studies include: spectrum bias (n = 15/15, or 100% of the studies, criterion 1), disease progression bias (n = 9/15, or 60%, criterion 4), incorporation bias (n = 1/15, or 7% of the studies, criterion 7), test review bias (n = 3/15, 20% of the studies, criterion 10), reference review bias (n = 1/15, 7% of the studies, criterion 11), and clinical review bias (n = 14/15, 93% of the studies, criterion 12). Details were insufficient on the following:

● how uninterpretable test results were handled (n = 14/15, 93% of the studies, criterion 13).

● information on selection criteria (n = 5/15, 33% of the studies, criterion 2)

● classification of target condition (2/15, 13 % of the studies, and criterion 3),

● procedure used for clinical examination (n = 15/15, or 100% of the studies, criterion 9) and

● procedure used in musculoskeletal ultrasound (n = 1/15, 7% of the studies, criterion 8).

### Descriptions of included studies

The characteristics of the 15 included studies and the reference sample population are reported in Additional file 1: Appendix 7. Description of diagnostic studies. Considering these included studies, fourteen [14] were published since 2000, potentially reflecting advances in musculoskeletal ultrasound technology, and an increasing focus on diagnostic validity studies.

In this review, the elbow with Lateral epicondylalgia was described as the symptomatic elbow. For individuals with one Lateral epicondylalgia elbow, the non-Lateral epicondylalgia elbow was the asymptomatic elbow. The elbows of participants who did not have Lateral epicondylalgia on either elbow were described as non-symptomatic.

Twelve (12) of the 15 studies compared the musculoskeletal ultrasound results of:

● symptomatic vs asymptomatic elbows [23,31];

● symptomatic vs non-symptomatic elbows [24,32-36] and

● symptomatic vs combined asymptomatic and non-symptomatic elbows [22,37-39].

Different brands of musculoskeletal ultrasound machines with frequencies of transducer heads ranging from 5 to 19 MHz were used in the included studies. Four studies used Colour Doppler Ultrasonography [23,32,35,40] and three studies [33,36,37] used Power Doppler Ultrasonography to detect neovascularity in the common extensor origin. One study used Real-Time Sonoelastrographic scanner to assess for the compressibility of the common extensor origin [22].

During the scanning procedure, elbows were either extended [33] or flexed [22-24,32,34-41] with the forearm either pronated [32,34,35,37,40], supinated [23] or in neutral position [22,23,33,34,38]. Two studies did not report on the position of the elbows during scan [31,42]. The images were scanned by radiologists in 10 studies [22,23,32-35,38,40-42], sonographers in two studies [31,37], radiologist and sonographer in one study [36], radiologist and body imager in one study [39] and orthopaedic surgeon in one study [24].

There were 666 patients included in the 15 studies; of who 297 were males (45.6%) and 369 were females (55.4%) Ages ranged from 16 to 70 years. Maffulli et al. (1990) [42] tested the youngest age group who were composed of tennis players (16–36 years old). The reported mean duration of elbow symptoms in 12 studies was more than 6 weeks [22,24,31,32,34-37,39-42] making Lateral epicondylalgia presentation of a chronic nature. One study [33] did not specifically identify the duration of symptoms but attributed most of their musculoskeletal ultrasound findings to chronicity of Lateral epicondylalgia. Two studies did not indicate their participants’ duration of elbow symptoms [23,38].

Twelve of the 15 studies [31-42] indicated the reference population from which their participants were drawn. In these studies, the patients were recruited from hospitals [32,33,35], local community [34], clinics [37,40], outpatients [31,41], self-referred [37], or referred by health care practitioners [33-40] or were tennis players [42]. The comorbidities and treatment of included patients were variably reported across all included studies.

In nine studies which reported the number of case and control participants [22,24,32-36,38,39], there was a total of 270 patients with Lateral epicondylalgia (128 males, 142 females) compared to 259 healthy participants (91 males, 168 females). The age range of patients with Lateral epicondylalgia (min-max: 13–70 years) was comparable to the healthy group (min-max: 17–71 years).

### Diagnostic value of the tests

For six studies, 2×2 contingency tables could not be constructed because of the following issues:

● A control group was lacking [40-42].

● Musculoskeletal Ultrasound findings for the control were not reported [23,36].

● Only over-all diagnostic sensitivity and specificity and diagnostic odds ratio were reported [38].

Additional file 1: Appendix 8. Sensitivity and Specificity of MSUS findings in elbows LE lists the diagnostic sensitivity and specificity for each musculoskeletal ultrasound technique and musculoskeletal ultrasound finding in each study from which statistics could be extracted. Studies which reported scanning of asymptomatic and non-symptomatic elbows but did not report findings on diagnostic specificity were labeled as not reported. Studies which did not investigate on the diagnostic specificity of asymptomatic and non-symptomatic elbows were labeled as not applicable.

Studies utilizing the same criteria in determining an abnormal musculoskeletal ultrasound finding are grouped as Criteria (e.g. A or B) and are reported in Additional file 1: Appendix 9. Criteria used to determine abnormal MSUS findings. Other variables that were common between the 15 diagnostic studies are reported in Additional file 1: Appendix 10. Similarities of collected MSUS data in 15 diagnostic studies. Common across all 15 studies were: a. the use of provocation tests as basis for recruitment; b. the use of transducer heads whose frequencies ranged between 5–15 MHz; c. participants with mean age between 30–55 years; and d. qualified interpreters of images.

Table 1 reports the pooled sensitivity and specificity of the musculoskeletal ultrasound findings from the comparable subset of papers, including 95% CI, p-value and I-squared for heterogeneity and the number of investigations from which results were combined. Separate analysis on studies which added sonographic probe-induced tenderness [24,32], increased blood flow on common extensor origin [40] and those studies which only used provocation tests as part of the reference standard were performed. Studies whose diagnostic sensitivity and specificity cannot be pooled were labeled as not applicable. Table 1 reports that:

**Table 1:** Pooled diagnostic validity of musculoskeletal ultrasound abnormalities in elbows with LE

**MSUS findings**	**N = investigations**	**Sensitivity**	**p-value, I**^**2**^	**N = investigations**	**Specificity**	**p-value, I**^**2**^
Over-all GS changes	3 [25,28,31]	0.77 (0.69-0.84)	0.81,0	3 [25,28,31]	0.73 (0.66-0.80)^$^	0.08,61
PDU + GS changes	4 [26,27,31]	0.69 (0.64-0.73)	<0.0001,97	4 [26,27,31]	0.82 (0.76-0.86)^$^	<0.001,85
Hypoechogenicity (criterion A)	2 [25,30]	0.65 (0.56-0.73)	0.74, 0	0	NA	NA
Hypoechogenicity (criterion B using RTSE)	3 [16]	0.64 (0.55-0.73)	<0.0001,89	3 [16]	0.96 (0.91-0.99)^$^	<0.01,82
Hypoechogenicity (criterion A with probe)	3 [18,25,30]	0.64 (0.56-0.72)	0.80,0	2 [18,25]	0.82 (0.72-0.90)^$^	0.61,0
Calcifications	3 [26-28]	0.33 (0.28-0.38)	<0.0001,96	3 [26-28]	0.97 (0.94-0.99)^#^	0.16,45
Neovascularity (PDU)	2 [27,31]	0.26 (0.21-0.32)	<0.0001,98	2 [27,31]	1.00 (0.97-1.00)^$^	0.10,63
Thickness (criterion A)	2 [30,31]	0.42 (0.32-0.53)	<0.01,88	0	NA	NA
Thickness (criterion B)	4 [27,28]	0.51 (0.47-0.55)	<0.0001, 95	4 [27,28]	0.80 (0.75-0.84)^#^	<0.0001, 94
Enthesopathy	2 [25]	0.38 (0.29-0.47)	<0.0001, 98	0	NA	NA
Cortical irregularities (criterion A)	2 [28,30]	0.20 (0.14-0.29)	0.53,0	0	NA	NA
Cortical spurs (criterion A)	2 [30,34]	0.13 (0.07-0.21)	0.03, 78	0	NA	NA
Bone changes (cortical irregularities or spurs) (criterion A)	2 [27,31]	0.56 (0.50-0.62)	0.41,0	2 [27,31]	0.84 (0.78-0.88)^$^	<0.0001, 96
Cortical irregularities (criterion A with sonographic probe-induced tenderness)	3 [26,28,30]	0.20 (0.14-0.27)	0.79,0	2 [26,28]	0.96 (0.88-0.99)^#^	0.34,0
Partial tear	2 [30,35]	0.29 (0.12-0.27)	0.02,80	0	NA	NA
Full tear	2 [30,35]	0.02 (0.00-0.06)	0.14,55	0	NA	NA

*Key:* GS, Gray-scale, I^2^, Iconsistency-square test for homogeneity; LE, Lateral Epicondylalgia; MSUS, musculoskeletal ultrasound; N, number; NA, not applicable; PDU, Power Doppler Ultrasonography; RTSE, Real-time Sonoelastography; #, healthy elbows; $, combined asymptomatic and healthy elbows.

● Hypoechogenicity of the common extensor origin has the best combination of diagnostic sensitivity [Sensitivity: 0.64 (0.56-0.72)] and specificity [Specificity: 0.82 (0.72-0.90)].

● Bone changes on the lateral epicondyle [Sensitivity: 0.56 (0.50-0.62)] were moderately sensitive in confirming elbows with chronic Lateral epicondylalgia.

● Neovascularity [Specificity: 1.00 (0.97-1.00)], calcifications [Specificity: 0.97 (0.94-0.99)] and cortical irregularities [Specificity: 0.96 (0.88-0.99)] have strong specificity for chronic Lateral epicondylalgia.

● No sufficient evidence supported the use of Colour Doppler Ultrasonography, Power Doppler Ultrasonography, Real-time Sonoelastography and sonographic probe-induced tenderness in confirming the presence of chronic Lateral epicondylalgia.

Forest plots were constructed from the groups of studies which reported the same diagnostic criteria in determining abnormal musculoskeletal ultrasound findings. An example of one Forest plot for over-all Gray-scale changes is presented in Figure 2. The remaining Forest plots are presented in Additional file 1: Appendix 11. Forest Plots of on Diagnostic Validity of Abnormal MSUS findings, Figures S3–S16.

**Figure 2:**
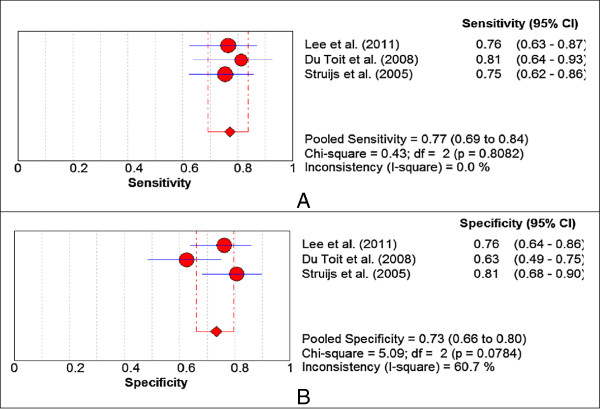
**Forest plots for over-all gray-scale changes. A**. Sensitivity, **B**. Specificity.

### High frequency (9–17 MHz) versus low frequency range (5–15 MHz)

The following studies were excluded from the analysis in determining the ability of the two frequency ranges in detecting abnormal musculoskeletal ultrasound findings in elbows with Lateral epicondylalgia. The reasons were as follows:

● Obradov and Anderson [32] utilised sonographic probe-induced tenderness to confirm an musculoskeletal ultrasound finding which could have influenced the final diagnosis given.

● Noh et al. [24] did not report the frequency of the transducer head used.

● De Zordo et al. [22] used Real-Time Sonoelastography to scan elbows with Lateral epicondylalgia.

Comparing the nearness of the pooled diagnostic sensitivity and specificity of the abnormal musculoskeletal ultrasound findings in Table 1 with the pooled diagnostic sensitivity and specificity of the abnormal musculoskeletal ultrasound findings based on the frequency of the transducer head used in Table 2, the following trends were observed:

**Table 2:** Diagnostic sensitivity and specificity based on frequency of the transducer head

	**High frequency**		**Low frequency**	
**MSUS findings**	**N**	**Sns,p-value,I**^**2**^	**N**	**SnS, p-value,I**^**2**^
		**SpC, p-value,I**^**2**^		**SpC, p-value,I**^**2**^
Hypoechogenicity	2 [17,28]	0.35(0.22-0.50), 0.0001,93	5 [25,29,30,33]	0.71(0.63-0.77),0.0009,79
		NA		NA
Calcifications	3 [17,27,28]	0.26(0.22-0.32),0.0001,89	4 [25,29,30,35]	0.11(0.07-0.16),0.0081,75
		NA		NA
Neovascularity	3 [17,27,31]	0.28(0.23-0.24),<0.00001,97	3 [29,30,34]	0.40(0.31-0.49),<0.0001,99
		100(98–100),0.25,28		NA
Thickness	4 [17,27,28,31]	0.53(0.49-0.57),<0.0001,93	3 [30,33,36]	0.27(0.21-0.33),0.0003,81
		NA		NA
Enthesopathy	1 [17]	0.64(0.24-0.91)	3 [25,35,36]	0.31(0.23-0.39),<0.0001,96
		NA		NA
Bone changes (cortical irregularities or bone spurs)	2 [27,31]	0.56(0.50-0.62),0.41,0	0	NA
		NA		NA
Cortical irregularities	1 [28]	0.18(0.08-0.31)	1 [30]	0.22(0.13-0.34)
		NA		NA
Cortical spurs	0	NA	2 [30,33]	0.13(0.07-0.21),0.03,78
		NA		NA
Partial tear	1 [17]	0.38(0.09-0.76)	2 [30,35]	0.06(0.02-0.11),0.10, 62
		NA		NA
Full tear	0	NA	2 [30,35]	0.02(0.00-0.06),0.14,55
		NA		NA

*Key*: I^2^, Inconsistency-square test for homogeneity; MSUS, musculoskeletal ultrasound; N, number; NA, not applicable; SnS, sensitivity; SpC, specificity.

● Low frequency transducer heads appear to detect hypoechogenicity and enthesopathy of the common extensor origin more frequently than high frequency transducer heads.

● High frequency transducer heads appear to detect calcifications and neovascularity of the common extensor origin more frequently than low frequency transducer heads.

● Based on the frequency of the transducer head used, there was not enough pool-able data determining the diagnostic specificity of abnormal musculoskeletal ultrasound findings.

Table 2 reports the pooling of diagnostic sensitivity and specificity (when applicable) of musculoskeletal ultrasound findings using high and low frequency ranges of transducer head.

### The reference standard

There were a number of ways in which a clinical diagnosis of Lateral epicondylalgia was used as reference standard. The Cozen test [22,24,31,33-35,37-42] was the most commonly used provocation test, and in five studies [31,33,35,38,40], this was the only test used to Lateral epicondylalgia. Other tests used (alone or in conjunction with others) were Maudsley [37,41,42], handgrip [39], sonographic probe-induced tenderness [32], resisted wrist supination [32], chair-lift test [34], and coffee-cup test [34]. Studies which used more than one provocation test did not report the sequence in which these tests were applied on the patients. In all included studies, the positions of the elbow and shoulder joints during provocation tests were not reported.

Other criteria used to clinically diagnose Lateral epicondylaliga were the following: a. surgical and histopathologic results [39], reduced grip strength [22,34,37], duration of lateral elbow pain [24,31,32,37,41] and previous sonographic findings [40].

## Discussion

This is the first known systematic review supporting the use of Gray-scale Ultrasonography as operated by qualified practitioners in detecting abnormal musculoskeletal ultrasound findings to confirm presence of Lateral epicondylalgia in individuals whose lateral elbow pain can be replicated by provocation test. There was insufficient evidence in the use of Colour Doppler Ultrasonography, Power Doppler Ultrasonography, Real-time Sonoelastography, sonographic probe-induced tenderness and high frequency transducer head to increase the diagnostic validity of musculoskeletal ultrasound in confirming Lateral epicondylalgia.

Hypoechogenicity of the common extensor origin has the best combination of diagnostic sensitivity and specificity being moderately sensitive and highly specific in determining elbows with Lateral epicondylalgia. Sonographically, hypoechogenicity can be detected on a normal background or in areas of degeneration and partial rupture. Hypoechogenicity of the common extensor origin, however, varies depending on the scanned area (anterior, middle, posterior sections) [22].

Bone changes on the lateral epicondyle were moderately sensitive to chronic Lateral epicondylalgia. Although cortical irregularities are suggested features of chronic stage of musculoskeletal disease [43-45], it is less frequently detected in Lateral epicondylalgia elbows compared to focal hypoechogenicity. This suggests a greater involvement of the common extensor origin in Lateral epicondylalgia.

Neovascularity, calcifications and cortical irregularities were strongly specific but poorly sensitive for chronic Lateral epicondylalgia. There is little clarity on the role of these findings in the diagnosis of Lateral epicondylalgia. Neovascularity is a vascular hyperplasia found in elbows with Lateral epicondylalgia [45]. It may be an infrequent indicator of the failed attempt of the common extensor origin to heal. However, the neovascularity in elbows with Lateral epicondylalgia does not contain patent lumens and does not correlate with improved healing [45,46]. Additionally, calcifications were inconsistently detected in elbows with Lateral epicondylalgia despite being considered a main feature of degenerative tendon changes [1,43]. This may indicate traumatic more than degenerative cause for the pathological changes in elbows with Lateral epicondylalgia.

There is no statistical evidence to recommend the use of Colour Doppler Ultrasonography, Power Doppler Ultrasonography, Real-time Sonoelastography, sonographic probe-induced tenderness and high frequency transducer heads in improving the diagnosis for Lateral epicondylalgia being that:

● Neovascularity is an inconsistent musculoskeletal ultrasound abnormality in elbows with Lateral epicondylalgia. Despite that neovascularity is commonly absent in healthy elbows (as indicated by its high specificity when using Power Doppler Ultrasonography), its diagnostic ability in confirming presence of Lateral epicondylalgia in elbows with pain has inconsistent results [34,36,37]. The diagnostic sensitivity and specificity of Real-time Sonoelastography is high yet comparable to the diagnostic sensitivity of Gray-scale Ultrasonography and diagnostic specificity of Colour Doppler Ultrasonography. Gray-scale Ultrasonography and Colour Doppler Ultrasonography are often practical to use in the clinical setting [17].

● The diagnostic utility of sonographic probe-induced tenderness may just be limited to replication of elbow pain without sufficient evidence of increasing the accuracy of locally identifying the site of abnormality in elbows of individuals with Lateral epicondylalgia.

Based on the mean age of participants, and duration of Lateral epicondylalgia symptoms reported in the papers included in this systematic review, we recommend the use of the pooled results for sensitivity and specificity of musculoskeletal ultrasound abnormalities as guide in objectively determining Lateral epicondylalgia in individuals aged between 16–70 years, with chronic Lateral epicondylalgia.

## Conclusion

The use of Gray-scale Ultrasonography (with 5-17 MHz transducer head) without sonographic probe-induced tenderness in detecting hypoechogenicity of the common extensor origin in elbows with pain was moderately sensitive [Sensitivity: 64 (56-72)] and highly specific [Specificity: 82 (72-90)] in determining elbows with Lateral epicondylalgia. The use of Power Doppler Ultasonography and Real-time Sonoelastography is expensive, and the evidence found in the review suggested that this technology did not significantly add to the sensitivity and specificity of Gray-scale Ultrasonography in detecting abnormal musculoskeletal findings in elbows with pain.

## Abbreviations

Asx: Asymptomatic; CSA: Cross sectional area; Df: Degrees of freedom; ECRB: Extensor carpi radialis brevis; EDC: Extensor digitorum communis; I^2^: Inconsistency square test for homogeneity; LE: Lateral epicondylalgia; MHz: Megahertz; MSUS: Musculoskeletal ultrasound; NA: Not applicable; NR: Not reported; NSx: Non-symptomatic; PDU: Power Doppler ultrasonography; PRISMA: Preferred reporting items for systematic reviews and meta-analyses; Q: Chi-square statistics with number of studies; QUADAS: Quality assessment for diagnostic accuracy studies; RTSE: Real-time sonoelastography; SnS: Sensitivity; SpC: Specificity; STARD: Standards for reporting of diagnostic accuracy.

## Competing interests

We certify that no party having a direct interest in the results of the research supporting this article has or will confer a benefit on us or on any organization with which we are associated AND, if applicable, we certify that all financial and material support for this research and work are clearly identified in the title page of the manuscript. The authors declare that they have no competing interests.

## Authors’ contribution

The following authors are listed based on their contributions. Conception and design of study: VCDIII, KG. Analysis and interpretation of data: VCDII, KG, KT, CGS, JL. Writing of the manuscript: VCDIII, KG, KT, CGS, JL. All authors read and approved the final manuscript.

## Pre-publication history

The pre-publication history for this paper can be accessed here:

http://www.biomedcentral.com/1471-2342/14/10/prepub

## Supplementary Material

Additional file 1 **Appendix 1.** QUADAS checklist. **Appendix 2**. STARD checklist. **Appendix 3**. Search results. **Appendix 4**. NHMRC Hierarchy of Evidence. **Appendix 5**. STARD Grades. **Appendix 6**. Quality Assessment for Diagnostic Accuracy Studies (QUADAS) scores. **Appendix 7**. Description of diagnostic studies. **Appendix 8**. Sensitivity and Specificity of MSUS findings in elbows LE. **Appendix 9**. Criteria used to determine abnormal MSUS findings. **Appendix 10**. Similarities of collected MSUS data in 15 diagnostic studies. **Appendix 11**. Forest Plots of on Diagnostic Validity of Abnormal MSUS findings. Click here for file
