# Supplementary material for: The diagnostic validity of musculoskeletal ultrasound in lateral epicondylalgia: a systematic review
Source: BMC Med Imaging. 2014 Mar 3;14:10. doi: 10.1186/1471-2342-14-10 (PMC4015882; doi:10.1186/1471-2342-14-10)
Supplement: Additional file 1 — Appendix 1. QUADAS checklist. Appendix 2. STARD checklist. Appendix 3. Search results. Appendix 4. NHMRC Hierarchy of Evidence. Appendix 5. STARD Grades. Appendix 6. Quality Assessment for Diagnostic Accuracy Studies (QUADAS) scores. Appendix 7. Description of diagnostic studies. Appendix 8. Sensitivity and Specificity of MSUS findings in elbows LE. Appendix 9. Criteria used to determine abnormal MSUS findings. Appendix 10. Similarities of collected MSUS data in 15 diagnostic studies. Appendix 11. Forest Plots of on Diagnostic Validity of Abnormal MSUS findings. [file 1471-2342-14-10-S1.pdf]

Appendix 1. QUADAS checklist

First Author: **Assessor:**
Date: **Journal:**

	Item	Yes	No	?
1	Was the spectrum of patients representative of the patients who will receive the test in practice? (Spectrum composition bias)			
Comment: Do info but probably patients in orthopaedic surgery clinic				
2	Were selection criteria clearly described?			
Comment:				
3	Is the reference standard likely to correctly classify the target condition?			
Comment:				
4	Is the time period between reference standard and index test short enough to be reasonably sure that the target condition did not change between the two tests? (Disease progression bias)			
Comment:				
5	Did the whole sample or a random selection of the sample, receive verification using a reference standard of diagnosis? (Partial verification bias)			
Comment:				
6	Did patients receive the same reference standard regardless of the index test result?			
Comment:				
7	Was the reference standard independent of the index test (i.e. the index test did not form part of the reference standard)? (Incorporation bias)			
Comment:				

8	Was the execution of the index test described in sufficient detail to permit replication of the test?			
Comment:				
9	Was the execution of the reference standard described in sufficient detail to permit its replication?			
Comment:				
10	Were the index test results interpreted without knowledge of the results of the reference standard? (Test review bias)			
Comment:				
11	Were the reference standard results interpreted without knowledge of the results of the index test?<br>(Reference review bias)			
Comment:				
12	Were the same clinical data available when test results were interpreted as would be available when the test is used in practice?<br>(Clinical review bias)			
Comment: as per Whiting instructions				
13	Were uninterpretable/ intermediate test results reported?			
Comment: no mention				
14	Were withdrawals from the study explained?			
Comment: all completed				
	**Total score**<br>Yes = 1 No/? = 0	9	4	1
Comments:				

References: QUADAS from Whiting et al., 2003; Bias categories from Fontela et al., 2009
Key: QUADAS, Quality Assessment for Diagnostic Accuracy Studies

Appendix 2. STARD Checklist

Source and Extraction

Author		Reference test	
Year		Index test	
Extractor		Country	
Multicentre?			
Comments:			

Was the article identified as a study of diagnostic accuracy (recommend MeSH heading sensitivity and specificity?	Yes	No

STARD QUESTION #1. If….	
Yes	Well covered
No	Not addressed

Primary aims	Secondary aims

STARD QUESTION #2.		
Was determining the diagnostic accuracy set as:		If yes, grade as:
a. Primary research aim	Yes/No	Well covered
b. Secondary research aim	Yes/No	Adequately addressed
Was the research aim to determine correlation of results rather than determining the diagnostic accuracy?	Yes/No	Poorly addressed

Inclusion Criteria	Exclusion Criteria
	1.

Were patients recruited from:	Check if related to the study
Private hospitals	
Public hospitals	
Clinics	
Sports centres	
General population	
Not indicated:	
Others:	

STARD QUESTION #3. If….	Grade as:
Inclusion criteria/exclusion criteria/setting were given…..	Well covered
Inclusion criteria/exclusion criteria were given….	Adequately addressed
Exclusion criteria were not mentioned	Poorly addressed
Participants were simply stated as being referred by general practitioners/orthopaedic surgeons …. For this paper, the inclusion/exclusion criteria were based on MRI findings	Poorly addressed

Recruitment period	Start to end (month/year)

STARD QUESTION #14. If….	Grade as
Beginning and end of recruitment were indicated, then….	Well covered
If none was given, then….	Not addressed

Was recruitment based on:	Check if related to the study:
Presenting symptoms	
Results from previous tests outside of the study	
Results from tests conducted during the study	

Were the participants recruited:	Check if related to the study.
Consecutively	
Non-consecutively	
Prospectively	
Retrospectively	

STARD QUESTION #4. If….	Grade as:
Either one of these was mentioned: recruitment was based on presenting symptoms, results from previous tests outside of the study, form tests conducted as part of the study	Well covered
Simply stated that recruitment was based on symptoms of lateral epicondylitis	Poorly addressed
Implied or not directly stated	Poorly addressed

STARD QUESTIONS #5,6. If….	Grade as
Type of recruitment (consecutive/non-consecutive) was specified, then….	Well covered
Type of data collection (prospective, restrospective) was specified, then….	Well covered
Type of recruitment was not specified, then….	Not addressed
Type of data collection was not specified, then….	Not addressed

What was the reference standard used in the study?	Check if related to the study:
Activities exacerbating the pain	
Previous medical diagnosis of lateral epicondylitis	
Tenderness on the lateral epicondyle	
Tenderness surrounding the lateral epicondyle	
Cozen's test	
Mill's test	
Maudsley's test	
Handgrip test	
Others: please specify.	Clinical findings

Rationale for use of reference standard	1.<br><br>2.<br><br>3.<br><br>4.

STARD QUESTION #7. If….	Grade as
Reference standard and rationale were stated, then….	Well covered
The reference standard was identified, then….	Adequately addressed
The reference standard was just implied, then….	Poorly addressed
Reference standard was not mentioned, then….	Not addressed

Musculoskeletal Ultrasound (MSUS)		Check the cell if not mentioned
Brand of MSUS machine		
Frequency of MSUS machine		
Frequency of transducer head		
If Doppler Imaging was used		
Pulsed repetition frequency		
Wall filter		
Colour gain		

	Please encircle appropriate answer.	Please specify range in which joints were positioned during scan.
Shoulder	Flexed<br><br>Extended	ROM:<br><br>ROM:
Elbow	Flexed<br><br>Extended<br><br>Neutral<br><br>Pronated<br><br>Supinated	ROM:<br><br>ROM:<br><br><br><br>ROM:<br><br>ROM:
Wrist	Neutral<br><br>Flexed<br><br>Extended	 <br>ROM:<br><br>ROM:
Not mentioned		

What pressure was used by the sonographer in scanning?	Mild pressure<br><br>Moderate pressure<br><br>Heavy pressure<br><br>Not mentioned

STARD QUESTION #8. If….	Grade as
The following were all identified:<br>a. Provocation tests used for the reference standard<br>b. At least one of the technical specifications of the ultrasound machine<br>OR At least one of the technical specifications of the probe<br>c. The position of the participants during MSUS	Well covered
The following were identified:<br>a. Provocation tests used for the reference standard<br>b. At least one of the technical specifications of the ultrasound machine<br>OR At least one of the technical specifications of the probe	Adequately addressed
The reference standard was broadly referred to as common tests used in diagnosing LEP	Poorly addressed
At least one of the technical specifications of the ultrasound machine	Poorly addressed
Technical specifications were not mentioned	Not addressed

Kindly write on the blank specific MSUS findings like cortical irregularities, spur, hypoechogenicity, etc.)	Subjective (if merely denotes presence or absence of)<br><br>List the findings:	Objective (if measurements were used)<br><br>List the findings:

STARD QUESTION #9. If….	Grade as
Findings on reference standard and index tests were given, then….	Well covered
Findings on index tests were given, then….	Adequately addressed
If findings did not directly determine the diagnostic accuracy of the index test, then….	Poorly addressed
If none was given, then….	Not addressed

MSUS findings	Diagnostic accuracy tests (Please specify if sensitivity, specificity, positive likelihood ratio, negative likelihood ratio)	Percentage	95% confidence interval	Comments

Other comments:

STARD QUESTION #12. If….	Grade as
Results on diagnostic accuracy and its 95% CI were given, then….	Well covered
Results on diagnostic accuracy without the 95% CI were given, then….	Adequately addressed
Based from raw data, the diagnostic accuracy can be computed, then….	Poorly addressed
If none was given, then….	Not addressed

STARD QUESTION #21. If….	Grade as
Estimates of diagnostic accuracy and measures of statistical uncertainty were given, then….	Well covered
Estimates of diagnostic accuracy were given, then	Poorly addressed
If none was given, then….	Not addressed

Fill up the 2X2 table. Kindly put on the blank the MSUS feature being tested for diagnostic accuracy.

Number of patients with ___________ and with elbow pain	Number of patients with ___________ and without elbow pain
Number of patients without_____________ and with elbow pain	Number of patients without ___________ and without elbow pain

STARD QUESTION #19. If….	Grade as
Cross tabulation of reference standard and index test was shown, then….	Well covered
Cross tabulation was not shown but diagnostic accuracy could be derived from data given in the study and computed by the authors, then….	Adequately addressed
If none was given, then….	Not addressed

Expertise of the testers

	Reference standard	Index test
Profession of tester		
Years of training of tester		
Profession of reader (if applicable)		
Years of training of reader (if applicable)		

STARD QUESTION #10. If….	Grade as
Years of training of testers for reference standard and index tests were given, then….	Well covered
Years of training of tester for index test were given, then….	Adequately addressed
Years of training of tester for reference standard were given, then….	Poorly addressed
Simply stated that the testers were trained, then….	Poorly addressed
If none was given, then….	Not addressed

	Reference standard	Index test
Tester was BLINDED to laterality of symptoms or results of previous examinations.		
Tester was NOT BLINED to laterality of symptoms or results of previous examinations.		

STARD QUESTION # 11. If….	Grade as
The sonographer was blinded to the results of previous examinations of the participant when interpreting index test (MSUS results), then….	Well covered
The interpreter was blinded to the results of previous examinations of the participant when interpreting index test (MSUS results), then….	Well covered
(If the sonographer and interpreter are different) If the interpreter during interpretation of the participant's MSUS results was blinded to the results of previous examinations of the participant, then….	Adequately addressed
(If the sonographer and interpreter are different) If the sonographer was blinded to the results of previous examinations of the participant, then….	Poorly addressed
This aspect of this study was ignored, then….	Not addressed

Reliability of Testers

Tester	MSUS findings	Results on Reliability	95% CI on reliability

STARD QUESTION # 13. If….	Grade as
Results on intra-tester reliability (if study has one tester only) was given, then….	Well covered
Results on intra-tester and inter-tester reliability (if study has two or more testers) were given, then….	Well covered
When one of the intertester/intratester reliability (if study has two or more testers) was given, then….	Adequately addressed
If none was given, then….	Not addressed

STARD QUESTION #24. If….	Grade as
95% CI or SD was given for reliability tests, then….	Well covered
(Applicable to two or more testers) 95% CI or SD was missing for either one of intra or intertester reliability, then….	Adequately addressed
If none was given, then….	Not addressed

Demographics

	Total	Male	Female
Number			
Number who underwent sonography			
Age			
Duration of elbow symptoms			
Number of symptomatic elbows			
Number of asymptomatic elbows			
Severity of symptoms (i.e. VAS)			
Current treatment			
Other:			

**Number of Excluded patients**	
LE group	
Healthy group	

Healthy participants	Total	Male	Female
Number			
Age			
Others:			

STARD QUESTION #15. If….	Grade as
Age, sex, duration of symptoms, number of symptomatic and asymptomatic elbows, severity of symptoms AND current treatment were given, then….	Well covered
Age, sex, duration of symptoms and number of symptomatic and asymptomatic elbows were given, then….	Adequately addressed
Not covered in categories well covered and adequately addressed, then….	Poorly addressed
If none was given, then….	Not addressed

Drop-outs

	Reference standard	Index test
Number of participants that did not undergo tests	0	1

STARD QUESTION #16. If….	Grade as
The drop-outs were not discussed explicitly due to absence of drop-outs, then….	Well covered
The number of drop-outs and reasons behind dropping-out for index test were indicated, then….	Well covered
The number of drop-outs of index test was indicated, then….	Poorly addressed
Not mentioned or indicates that this aspect of the study was ignored, then….	Not addressed

STARD QUESTION #22. If….	Grade as
There were no drop-outs/outliers/missing responses based on flow of the study (even without stating zero to report drop-outs/outliers/missing responses), then….	Well covered
drop-outs/outliers/missing responses were indicated and inclusion/exclusion in the analysis was defended, then….	Well covered
drop-outs/outliers/missing responses were indicated and inclusion/exclusion in the analysis was not explained, then….	Poorly addressed
Drop-outs/outliers/missing responses were not indicated	Not addressed

What is the time interval between provocation tests and MSUS?	
Comments	

STARD QUESTION # 17. If….	Grade as
Time interval between reference standard and index test was indicated, then….	Well covered
The time interval between the reference standard and index test was implied and not explicitly stated, then….	Poorly addressed
Not mentioned or indicates that this aspect of the study was ignored, then….	Not addressed

Were the participants categorised according to severity of disease?	Yes	No

STARD QUESTION #18. If….	Grade as
Severity of disease was categorised for participants with LEP, then….	Well covered
Categories on severity of disease were not made, then….	Not addressed

STARD QUESTION #23. If….	Grade as
Estimates of variability of diagnostic accuracy between subgroups of participants, readers or centers, if done	Well covered
Estimates of variability of diagnostic accuracy between subgroups of participants, readers or centers, if NOT done	Not addressed

		Reference standard	Index test
Adverse effects of tests		1.<br>2.<br>3.<br>4.<br>5.	1.<br>2.<br>3.<br>4.<br>5.

STARD QUESTION #20. If….	Grade as
Adverse events for index test were indicated, then….	Well covered
Adverse events were not mentioned….	Not addressed

What is the clinical applicability of the study findings?

STARD QUESTION #25. If….	Grade as
Discussed appropriate to the results obtained, then….	Well covered
Discussed but not fully supported by the results obtained, then….	Poorly addressed
Not mentioned or indicates that this aspect of the study was ignored, then….	Not addressed

Key: STARD, Standards for Reporting of Diagnostic Accuracy

Appendix 3. Search results

Database	No	Keyword	Hits
**OVID**<br><br>(AMED, Books@Ovid, Journals@Ovid Full Text, EMBASE, ICONDA, OVID Medline, Ovid Nursing database, Your Journals@Ovid, The Joanna Briggs Institute EBP Database)	1	Lateral epicondylitis OR tennis elbow OR radial epicondyaligia OR lateral epicondylalagia OR extensor tendinopath* OR epicondylitis lateralis humeris OR lateral elbow tendinopath* OR lateral epicondylosis OR lateral tennis elbow	6,617
	2	Limit #1 to 1990-Current	5,573
	3	Sonography OR ultrasound OR musculoskeletal ultrasound OR diagnostic ultrasound	782,783
	4	Limit #3 to 1990-Current	710,393
	5	Sensitivity OR specificity OR diagnostic accuracy OR diagnosis OR accuracy	9,765,039
	6	Limit #5 to 1990-Current	7,261,585
	7	1+3+5	650
	8	Limit #7 to 1990-Current	643
	9	De duplicate #8	517

Database	No	Keyword	Hits
**EBSCO**<br><br>(Academic Search Premier, Ageline, CINHAL, E-journals, Ergonomics Abstracts, ERIC, Health Source-Consumer edition, Health Source-Nursing Academic edition, PsycArticles, PsycBOOKS, SPORTDiscus)	1	lateral epicondylitis OR tennis elbow OR radial epicondyl* OR lateral epicondyl* OR extensor tendinopath* OR epicondylitis lateralis humer*s OR lateral elbow tendinopath* OR lateral tennis elbow	4,530
	2	Limit 1 from 1990-2013	4,081
	3	Sonography OR ultrasound OR musculoskeletal ultrasound OR diagnostic ultrasound	266,962
	4	Limit 3 from 1990-2013	257,867
	5	sensitivity OR specificity OR diagnostic accuracy OR diagnosis OR accuracy OR ROC OR false positive OR false negative OR predictive value$ OR likelihood ratio$ OR reference values OR reference standards	2,887,388
	6	Limit 5 from 1990-2013	2,669,690
	7	1 + 3 + 5	102
	8	2 + 4 + 6	101

Database	No	Keyword	Hits
**COCHRANE**	1	Lateral epicondyl* OR tennis elbow OR radial epicondyla* OR lateral epicondyla* OR extensor tendinopath* OR epicondylitis lateralis humeris OR lateral elbow tendinopath* OR lateral epicondylosis OR lateral tennis elbow	357
	2	Limit 1 from 1990-2013	329
	3	Sonography OR ultrasound OR musculoskeletal ultrasound OR diagnostic ultrasound	7,551
	4	Limit 3 from 1990-2013	7,140
	5	sensitivity OR specificity OR diagnostic accuracy OR diagnosis OR accuracy OR ROC OR false positive OR false negative OR predictive value$ OR likelihood ratio$ OR reference values OR reference standards	68,382
	6	Limit 5 from 1990-2013	62,095
	7	1+3+5	1,197
	8	2+4+6	1,093

Database	No	Keyword	Hits
**Web of knowledge**	1	Lateral epicondylitis OR tennis elbow OR radial epicondyaligia OR lateral epicondylalagia OR extensor tendinopath* OR epicondylitis lateralis humeris OR lateral elbow tendinopath* OR lateral epicondylosis OR lateral tennis elbow (1950-2012)	4,574
	2	Limit 1 from 1990-2013	4,086
	3	Sonography OR ultrasound OR musculoskeletal ultrasound OR diagnostic ultrasound (1950-2012)	514,513
	4	Limit 3 from 1990-2013	481,298
	5	"sensitivity and specificity" OR "sensitivity and specificity/standards" OR "specificity" OR “screening” OR “false positive” OR "false negative" OR "accuracy" OR "predictive value" OR “predictive value of tests" OR “predictive value of tests/standards" OR "predictive values" OR “predictive values of tests” OR “reference value” OR “reference values” OR “reference values/standards” OR “roc” OR “roc analyses” OR “roc analysis” OR “roc and” OR “roc area” OR “roc auc” OR “roc characteristics” OR “roc curve” OR “roc estimated” OR “roc evaluation” OR “likelihood ratio” OR “diagnostic accuracy” (1950-2012)	3,898,364
	6	Limit 5 from 1990-2013	3,502,131
	7	1+3+5	21
	8	2+4+6	21

Database	No	Keyword	Hits
**Web of Science**	1	Lateral epicondylitis OR tennis elbow OR radial epicondyaligia OR lateral epicondylalagia OR extensor tendinopath* OR epicondylitis lateralis humeris OR lateral elbow tendinopath* OR lateral epicondylosis OR lateral tennis elbow (1983-2013)	1,400
	2	Sonography OR ultrasound OR musculoskeletal ultrasound OR diagnostic ultrasound (1983-2013)	202,994
	3	Sensitivity OR specificity OR diagnostic accuracy OR diagnosis OR accuracy (1983-2013)	2,163,053
	4	1+2+3	46
	5	Limit #4 to 1999-2013	46
**Science Direct**	1	Lateral epicondylitis OR tennis elbow OR radial epicondyaligia OR lateral epicondylalagia OR extensor tendinopath* OR epicondylitis lateralis humeris OR lateral elbow tendinopath* OR lateral epicondylosis OR lateral tennis elbow (1823-present)	103
	2	Limit 1 from 1990-2013	102
	3	Sonography OR ultrasound OR musculoskeletal ultrasound OR diagnostic ultrasound (1823-present)	345,779
	4	Limit 3 from 1990-2013	314,962
	5	Sensitivity OR specificity OR diagnostic accuracy OR diagnosis OR accuracy (1823-present)	1,201.090
	6	Limit from 1990-2013	992,682
	7	1+3+5	29
	8	2+4+6	29

Database	No	Keyword	Hits
**PubMed**	1	Lateral epicondyl* OR tennis elbow OR radial epicondyla* OR lateral epicondyla* OR extensor tendinopath* OR epicondylitis lateralis humeris OR lateral elbow tendinopath* OR lateral epicondylosis OR lateral tennis elbow	1,909
	2	Limit 1 from 1990-2013	1,528
	3	Sonography OR ultrasound OR musculoskeletal ultrasound OR diagnostic ultrasound	412,343
	4	Limit 3 from 1990-2013	336,133
	5	sensitivity OR specificity OR diagnostic accuracy OR diagnosis OR accuracy OR ROC OR false positive OR false negative OR predictive value* OR likelihood ratio* OR reference value* OR reference standard*	8,846,967
	6	Limit from 1990-2013	6,031,537
	7	1+2+3	98
	8	2+4+6	96

Database	No	Keyword	Hits
**HIGH WIRE PRESS**	1	lateral epicondy* OR tennis elbow AND ultrasound AND sensitivity OR specificity OR diagnostic accuracy OR accuracy	29
	2	Limit 1 from 1990-2013	29
**GOOGLE SCHOLAR**	1	tennis elbow OR lateral epicondylitis AND sonography OR ultrasound OR diagnostic ultrasound OR musculoskeletal ultrasound AND sensitivity OR specificity	701
	2	Limit 1 from 1990-2013	648

Appendix 4. NHMRC Hierarchy of Evidence

Levels of Evidence	Diagnostic Accuracy	Articles
I	Systematic Review of level II studies	None
II	Independent, blinded comparison with a valid reference standard, among consecutive persons with a defined clinical presentation	Ultra sonographic findings for chronic lateral epicondylitis (Obradov and Anderson, 2012)<br><br>Common extensor tendon thickness measurements at the radiocapitellar region in diagnosis of lateral elbow tendinopathy (Toprak et al., 2012)<br><br>Utility of Sonographic Measurement of the Common Tensor Tendon in Patients with Lateral Epicondylitis (Lee et al., 2011)<br><br>Real-Time Sonoelastography of Lateral Epicondylitis: Comparison of Findings Between Patients and Healthy Volunteers (De Zordo et al., 2009)<br><br>Value of Ultrasonography on Diagnosis and Assessment of Pain and Grip Strength in Patients with Lateral Epicondylitis (Tarhan et al., 2009)<br><br>Diagnostic Accuracy of power Doppler US in patients with chronic tennis elbow (Du Toit et al., 2008)<br><br>Extensor origin vascularity related to pain in patients with tennis elbow (Zeisig et al., 2006)

Levels of Evidence	Diagnostic Accuracy	Articles
III-1	Independent, blinded comparison with a valid reference standard, among non-consecutive persons with a defined clinical presentation	A two-year sonographic follow-up after intra-tendinous therapy in patients with tennis elbow (Zeisig et al., 2010)<br><br>The predictive value of Diagnostic Sonography for the Effectiveness of Conservative Treatment of Tennis Elbow (Struijs et al., 2005)<br><br>Lateral Epicondylitis of the Elbow: US Findings (Levin et al., 2005)<br><br>Comparison of Sonography and MRI for Diagnosing Epicondylitis (Miller et al., 2002)<br><br>Tennis elbow: an ultrasonographic study in tennis players (Maffulli et al., 1990)
III-2	A comparison with reference standard that does not meet the criteria required for Level II and III-1 evidence	Sonographic probe induced tenderness for lateral epicondylitis (Noh et al., 2010)<br><br>“Tenomalacia”: a new sonographic sign of tendinopathy? (Khoury and Cardinal, 2009)<br><br>Sonographic examination of lateral epicondylitis (Connell et al., 2001)
III-3	Diagnostic case-control study	None
IV	Study of diagnostic yield (with no reference standard)	None

Key: NHMRC, National Health and Medical Research Council; SR, systematic review

Appendix 5. STARD Grades

		Obradov and Anderson	Toprak et al.	Lee et al.	Zeisig et al.	Noh et al.	de Zordo et al.	Khoury and Cardinal	Tarhan et al.	du Toit et al.	Zeisig et al.	Struijs et al.	Levin et al.	Miller et al.	Connell et al.	Maffulli et a.
		2012	2012	2011	2010	2010	2009	2009	2009	2008	2006	2005	2005	2002	2001	1990
**STARD**																
1	Title	0	1	0	0	0	0	0	0	1	0	0	1	1	0	0
2	Rationale	0	1	1	0	1	1	1	0	1	1	0	1	1	1	1
3	Eligibility criteria	1	1	1	1	1	1	0	1	1	1	1	0	1	1	1
4	Basis of recruitment	1	1	1	1	1	1	1	1	1	1	1	0	1	1	1
5	Consecutive recruitment?	1	0	1	0	1	1	1	1	1	1	1	0	0	1	0
6	Data collection process	1	1	1	1	1	1	1	1	1	1	1	1	1	1	1
7	Reference standard and rationale	1	1	1	0	0	1	0	0	1	0	0	1	1	0	0
8	Technical specifications	1	1	1	1	1	1	1	1	1	1	1	1	1	1	1
9	Definition of results	1	1	1	1	1	1	1	1	1	1	1	1	1	1	1
10	Training	1	1	1	1	1	1	1	1	1	1	1	1	1	1	0
11	Blinding	1	0	1	1	1	1	0	1	1	1	1	1	1	0	1

Key: STARD, Standards for Reporting of Diagnostic Accuracy; 0, poorly addressed or not addressed; 1, well covered or adequately addressed

		Obradov and Anderson	Toprak et al.	Lee et al.	Zeisig et al.	Noh et al.	de Zordo et al.	Khoury and Cardinal	Tarhan et al.	du Toit et al.	Zeisig et al.	Struijs et al.	Levin et al.	Miller et al.	Connell et al.	Maffulli et a.
		2012	2012	2011	2010	2010	2009	2009	2009	2008	2006	2005	2005	2002	2001	1990
**STARD**																
12	Statistics used	1	1	1	1	0	1	0	1	1	0	1	1	1	0	0
13	Reliability tests	0	0	1	0	0	0	0	0	0	0	0	1	0	0	0
14	Recruitment period	1	0	1	0	1	0	0	0	1	0	1	1	0	1	0
15	Demographics	1	1	1	1	1	1	1	1	1	1	1	1	1	1	1
16	Drop outs	1	1	1	1	1	1	1	1	1	1	1	1	1	0	1
17	Time interval	0	1	0	0	0	1	0	0	1	0	0	0	0	0	0
18	Severity of symptoms	0	0	0	0	1	1	0	1	0	0	0	0	0	0	0
19	Cross tabulation	1	1	1	0	1	1	0	0	1	1	1	0	0	0	0
20	Adverse events	0	0	0	0	0	0	0	0	1	0	0	0	0	0	0
21	Diagnostic Accuracy and 95% CI	1	0	1	0	0	0	0	0	1	0	0	1	0	0	0
22	Drop-outs	1	1	1	1	1	1	1	1	1	1	1	1	1	0	1
23	Subgroup analysis	0	1	0	0	0	0	0	1	1	0	0	0	0	0	0
24	Estimates for reliability	0	0	1	0	0	0	0	0	0	0	0	1	0	0	0
25	Clinical applicability	1	1	1	1	1	1	1	1	1	1	1	1	1	1	1

Key: STARD, Standards for Reporting of Diagnostic Accuracy; 0, poorly addressed or not addressed; 1, well covered or adequately addressed

Appendix 6. Quality Assessment for Diagnostic Accuracy Studies (QUADAS) scores

Authors, Year	1	2	3	4	5	6	7	8	9	10	11	12	13	14
Obradov and Anderson (2012)[26]	N	Y	Y	?	Y	Y	Y	Y	N	Y	Y	N	?	Y
Toprak et al. (2012)[27]	N	Y	Y	Y	Y	Y	Y	Y	N	?	Y	?	?	Y
Lee et al. (2011)[28]	N	Y	Y	N	Y	Y	Y	Y	N	Y	Y	N	?	?
Zeisig et al. (2010)[34]	N	N	Y	Y	Y	Y	N	Y	N	Y	N	N	?	Y
Noh et al. (2010)[18]	N	Y	Y	?	Y	Y	Y	Y	N	N	Y	Y	?	Y
de Zordo et al. (2009)[16]	N	Y	Y	Y	Y	Y	Y	Y	N	Y	Y	N	?	Y
Khoury and Cardinal (2009)[17]	N	N	N	?	Y	Y	Y	Y	N	?	Y	?	?	Y
Tarhan et al. (2009)[35]	N	Y	Y	?	Y	Y	Y	Y	N	Y	Y	N	?	Y
du Toit et al. (2008)[31]	N	Y	Y	Y	Y	Y	Y	Y	N	Y	Y	N	?	Y
Zeisig et al.(2006)[29]	N	Y	Y	Y	Y	Y	Y	Y	N	Y	Y	N	?	Y
Struijs et al. (2005)[25]	N	Y	Y	?	Y	Y	Y	Y	N	Y	Y	N	Y	Y
Levin et al. (2005)[32]	N	Y	Y	?	Y	Y	Y	Y	N	Y	Y	N	?	Y
Miller et al. (2002)[33]	N	N	Y	N	Y	Y	Y	Y	N	Y	Y	N	?	Y
Connell et al. (2001)[30]	N	N	?	Y	Y	Y	Y	Y	N	?	Y	?	?	N
Maffulli et al. (1990)[36]	N	N	Y	?	Y	Y	Y	N	N	Y	Y	N	?	Y

Key: N, No; QUADAS, Quality Assessment for Diagnostic Accuracy Studies; Y, Yes; ?, undetermined

Appendix 7. Description of diagnostic studies

Author, Year, Country	Selection of patients	Source of patients	Recruitment	Basis for recruitment	Reference standard	Reader/blinding	MSUS machine/frequency of transducer head
Obradov and Anderson (2012)[26], The Netherlands	*Inclusion Criteria*<br>(+)LE symptoms for 6 months<br>(+)sonographic probe induce tenderness<br><br>*Exclusion Criteria*<br>(+)previous surgery<br>(+)systematic joint disease	Hospital	Consecutive, retrospective	Presenting symptoms	Sonographic probe induced tenderness	2 Radiologists/blinded	ATL 5000, Philips, The Netherlands/5-12 MHz linear-array transducer
Toprak et al. (2012)[27], Turkey	*Inclusion Criteria*<br>(+)tenderness on<br>(+)Cozen<br><br>*Exclusion Criteria*<br>(+) NSAIDs within 3 weeks prior to the study<br>(+) surgery<br>(+) acute trauma	Hospitals Referred by physical rehabilitation and medical specialists	Consecutive, prospective	Presenting symptoms Results from previous test	Tenderness on lateral epicondyle<br>(+) Cozen's test	Radiologist/blinded	General electric medical systems, Milwaukee, Wisconsin, USA, 12 MHz (10-14 MHz) probes in real time

Author, Year, Country	Selection of patients	Source of patients	Recruitment	Basis for recruitment	Reference standard	Reader/blinding	MSUS machine/frequency of transducer head
Lee et al. (2011)[28], Korea	*Inclusion Criteria:*<br>(+)hx of LE<br>(+)s/sx of LE<br>(+) provisional diagnosis of LE<br><br>*Exclusion Criteria:*<br>(+)steroid injection < 3 weeks prior to MSUS<br>(+)hx of acute trauma or previous surgery<br><br>*Healthy Group:*<br>(-)S/Sx of LE<br>(-)hx of CTD<br>(-)inflammatory arthritis<br>(-)hx elbow injury	Local community<br><br>Referred by orthopaedic surgeons	Presenting symptoms<br><br>Results from previous tests	Presenting symptoms<br><br>Results from previous tests	Tenderness on lateral epicondyle<br>(+) Cozen's tests<br>Reduced grip strength<br>(+) chair-test<br>(+) coffee-cup test	Radiologist/blinded	ATL HDI 5000 UI-22 Philips Healthcare<br>linear array, 12 MHz

Author, Year, Country	Selection of patients	Source of patients	Recruitment	Basis for recruitment	Reference standard	Reader/blinding	MSUS machine/frequency of transducer head
Zeisig et al. (2010)[34], Sweden	*Inclusion Criteria:*<br>(+)tenderness<br>(+)Cozen<br>(+)high blood flow in CEO<br><br>*Exclusion Criteria:*<br>(+) RN compression	Sports Medicine Unit<br><br>Referred by hand surgeons	Non-consecutive, prospective	Presenting symptoms<br><br>Results from previous tests	(+)tenderness on lateral epicondyle<br>(+)Cozen<br>(+)increased blood flow in CEO	Radiologist/blinded	High resolution grey-scale US and CD, Accuson Sequoia 512<br><br>8-13 MHz
Noh et al. (2010)[18], Korea	*Inclusion Criteria:*<br>(+)LE >3 weeks<br>(+)VAS at rest >4<br>(+) conservative medical treatment<br>(-)hx of steroid injection<br>(-)hx of PT	Not indicated	Consecutive, prospective	Presenting symptoms	(+)lateral elbow pain (> 3 weeks)<br>(+)tenderness on lateral epicondyle<br>(+)Cozen's test<br>(+)sonographic probe induced tenderness	Orthopedic surgeon/not blinded	Accuvix XQ, Medison, Korea
De Zordo et al. (2009)[16], Austria	*Inclusion Criteria:*<br>(+)LE Sx<br><br>*Exclusion Criteria:*<br>(+)hx of tendon rupture<br>(+)hx of systematic inflammatory D/O<br><br>*Healthy group:*<br>(-) LE examination	Not indicated	Consecutive, prospective	Presenting symptoms	(+)lateral elbow pain<br>(+)tenderness on lateral epicondyle<br>(+)Cozen<br>(+)reduced grip strength	2 Radiologists: blinded	RTSE scanner<br>EUB 9000, EUP-154M, Hitachi Medical<br>6-13 MHz<br><br>Power Doppler MSUS<br>MyLab 90 scanner, Esaote linear array (LA 435, Esaote) at 6-18 MHz

Author, Year, Country	Selection of patients	Source of patients	Recruitment	Basis for recruitment	Reference standard	Reader/blinding	MSUS machine/frequency of transducer head
Khoury and Cardinal, (2009)[17], Canada	*Inclusion Criteria:*<br>(+)MSUS findings<br>(+)unilateral extensor tendinopathy	Not indicated	Consecutive, prospective	Presenting symptoms	Clinical diagnosis of LE	Radiologist/not indicated	iU22 system, Philips Medical Systems<br><br>15 MHz hockey stick transducer
Tarhan et al. (2009)[35], Turkey	*Inclusion Criteria:*<br>(+)intermittent pain for 3 weeks<br>(+)tenderness 2cm of lateral epicondyle<br>(+)Cozen<br>(+)Maudsley<br><br>*Exclusion Criteria:*<br>(+)hx elbow fracture<br>(+)congenital or acquired elbow deformities<br>(+)inflammatory rheumatic disorder	Outpatient	Consecutive, prospective	Presenting symptoms	(+)lateral elbow pain (at least 3 weeks)<br>(+)Cozen<br>(+)Maudsley<br>(+)exacerbated pain vs constant pain	Radiologist/blinded	Sonoline G50, Siemens, Seattle, WA, USA<br><br>linear array, 8-12 MHz

Author, Year, Country	Selection of patients	Source of patients	Recruitment	Basis for recruitment	Reference standard	Reader/blinding	MSUS machine/frequency of transducer head
du Toit et al. (2008)[31], Australia	*Inclusion Criteria:*<br>(+) lat elbow pain > 3mos.<br>(+)Cozen<br>(+) pain when gripping<br>(+)tenderness on lateral epicondyle<br><br>*Exclusion Criteria:*<br>(+)pain <3 mos.<br>(+)LOM of elbow<br>(+)upper neurological S/Sx<br>(+)corticosteriod injection <3 mos.<br><br>*Healthy group:*<br>(-)current or past Hx of lateral elbow pain<br>(-)tenderness<br>(+)full ROM<br>(+)pain-free grip	Private clinics referred by general practitioners, sports physicians self-referred	Consecutive, prospective	Presenting symptom<br><br>Results from previous tests	(+)lateral elbow pain (>3 months)<br>(+)tenderness on lateral epicondyle<br>(+)Cozen<br>(+) Maudsley<br>(+)reduced grip strength	Sonographer/blinded	Philips IU22 US machine<br>Grey-scale: 17-5 MHz<br>Doppler: Pulsed repetition frequency: 1000 Hz<br>Wall filter: 75 Hz<br>Colour gain: 86%

Author, Year, Country	Selection of patients	Source of patients	Recruitment	Basis for recruitment	Reference standard	Reader/blinding	MSUS machine/frequency of transducer head
Zeisig et al. (2006)[29], Sweden	*Inclusion Criteria:*<br>(+)tenderness<br>(+)Cozen<br><br>*Exclusion Criteria:*<br>(+)synovitis in proximal RU joint<br>(+)RN Entrapment<br>(+)arthritis	Public hospital<br><br>Referred by general practitioner	Consecutive, prospective	Presenting symptoms<br><br>Results from previous tests	(+)tenderness on lateral epicondyle<br>(+)Cozen	Radiologist/blinded	Acuson Sequoia 512<br>8-13 MHz<br><br>Colour Doppler velocity technique
Struijs et al. (2005)[25], The Netherlands	*Inclusion criteria:*<br>(+)clinical diagnosis of LE<br>(+)tenderness<br>(+)Cozen<br>(+)pain not <6 weeks<br><br>*Exclusion Criteria:*<br>(+)bilateral LE<br>(+) dec pain <2wks<br>(+) treatment in previous 6 mos.<br>Inability to fill out questionnaire	Outpatient	Non consecutive, retrospective	Presenting symptoms	(+)clinical diagnosis of LE<br>(+)tenderness<br>(+)Cozen<br>(+)pain not <6 weeks	Sonographer/ blinded	SSD 900 Aloka<br><br>7.5 MHz linear array

Author, Year, Country	Selection of patients	Source of patients	Recruitment	Basis for recruitment	Reference standard	Reader/blinding	MSUS machine/frequency of transducer head
Levin et al. (2005)[32], United States	*Inclusion Criteria:*<br>Referred by the sports medicine physician<br><br>*Healthy Group:*<br>Non-tender elbows<br>No history of elbow problems<br>Did not undergo clinical exam	Referred by Sports medicine physician	Non-consecutive, retrospective	Presenting symptoms<br><br>Results from previous tests	(+)tenderness on lateral epicondyle<br>(+)Cozen	2 Radiologists/blinded	Siemens Elegra, Siemens Medical Systems, Issaquah, Wash; or Philips HDI, 5000, Philips Medical Systems, Bothell, Wash<br><br>Multifrequency linear array with peak frequency of 12 or 13 MHz
Miller et al. (2002)[33], United States	*Inclusion Criteria:*<br>(+)elbow pain<br>(+)tenderness<br>(+)Cozen<br>(+)dx by orthopaedic surgeon<br><br>*Healthy group:*<br>No history of pain	Referred by orthopaedic surgeons	Non-consecutive, retrospective	Presenting symptoms<br><br>Results from previous tests	(+)lateral elbow pain<br>(+)tenderness on lateral epicondyle<br>(+)Cozen<br>(+)pain when gripping<br>(+)surgical and histopathologic confirmation	Radiologist and body imager/blinded	HDI 3000 US scanner (Advanced Technology Laboratories, Bothell, WA linear array or compact transducer, 5-10 MHz

Author, Year, Country	Selection of patients	Source of patients	Recruitment	Basis for recruitment	Reference standard	Reader/blinding	MSUS machine/frequency of transducer head
Connell et al. (2001)[30], Australia	*Inclusion Criteria:* Patients with lateral elbow pain	Referred by orthopaedic surgeons, rheumatologists and sports medicine physicians	Results from previous tests	Consecutive, prospective	Clinical diagnosis of LE	Sonographer and radiologist/not indicated	HDI 3000, ATL, Bothell, WA 10 MHz
Maffulli et al. (1990)[36], Italy		Tennis players	Presenting symptoms	Non-consecutive, retrospective	(+)lateral elbow pain (+)Cozen (+)Maudsley	2 Radiologists/blinded	Linear 7 MHz probe and a sector of 5 MHz probe

Key: CEO, common extensor origin; CTD, connective tissue disorder; dec, decreased; D/O, disorder; dx, diagnosed; Hx, history; LE, lateral epicondylalgia; MHz, Megahertz; MSUS, musculoskeletal ultrasound; mo(s), month(s); PT, physical therapy; RN, Radial Nerve; RTSE, Real-time Sonoelastography; RU, radioulnar; S/Sx, signs and symptoms; Sx, symptoms; USA, United States of America; VAS, Visual Analogue Scale

Appendix 7. Characteristics of studied population

Author, Year, Country	Number and gender of LE participants	Age of LE participants (mean, SD, range) in years	Number of symptomatic elbows	Duration of symptoms	Number and gender of healthy participants	Age of healthy participants (mean, SD, range) in years	Number of asymptomatic elbows
Obradov and Anderson (2012)[26], The Netherlands	43 (23 males, 20 female)	47(7.7)(NR)	49 (6 bilateral)	At least 6 mos.	5 (2 male, 3 female)	36(8.7)(NR)	10 (healthy)
Toprak et al. (2012)[27], Turkey	164 (54 male, 110 female)	43(NR)(19-66)	248 (84 bilateral, 144 dominant)	Categorised as chronic	80 (20 male, 60 female)	39(NR)(17-71)	160 (healthy)
Lee et al. (2011)[28], Korea	48 (11 male, 37 female)	48.3(NR)(34-66)	51 (3 with bilateral affectations)	3 months (2 wks - 6 mos.)	63 (15 male, 48 female)	48.3(NR)(39-63)	63 (healthy elbows)
Zeisig et al., (2010)[34], Sweden	25 (12 male, 13 female)	46(NR)(27-66)	28 (3 with bilateral affectations)	18 mos. (3-60 mos.)	NA	NA	N/A
Noh et al. (2009)[18], Korea	27 (12 male, 15 female)	44*(NR)(37-59)	27 (22 dominant, no bilateral affectation)	8.7 weeks (3-18 weeks)	27 (13 male, 14 female)	43*(NR)(37-59)	27 (healthy)

Author, Year, Country	Number and gender of LE participants	Age of LE participants (mean, SD, range) in years	Number of symptomatic elbows	Duration of symptoms (mean, SD, range)	Number and gender of healthy participants	Age of healthy participants (mean, SD, range) in years	Number of asymptomatic elbows
De Zordo et al. (2009)[16], Austria	32 (10 male, 22 female)	52.6(NR)(38-70)	38 (6 with bilateral affectations)	9 (24.36)(6-120) mos.	28 (11 male, 17 female)	43.6(NR)(24-89)	44 (12 asymptomatic elbows, 32 healthy elbows)
Khoury and Cardinal, (2009)[17], Canada	8 (1 male, 7 female)	45(NR)(NR)	8 (no bilateral affectation)	Not indicated	NA	NA	7 (asymptomatic elbows)
Tarhan et al. (2009)[35], Turkey	52 (13 male, 39 female)	Grp1: 49.8(9.6)NR Grp2: 44.8(8.9)NR	52 (49 dominant, no bilateral affectations)	8.2*(NR) (1-72) mos.	NA	NA	NA
du Toit et al. (2008)[31], Australia	25 (15 male, 10 female)	50(9)NR	32 (11 dominant, 7 with bilateral affectation)	10(NR)(3-120) mos.	19 (9 male, 10 female)	45(10)(NR)	56 (18 asymptomatic and 38 healthy elbows)
Zeisig et al. (2006)[29], Sweden	17 (7 male, 10 female)	45(NR)(NR)	22 (5 with bilateral affectations)	18 (NR)(NR) mos.	11 (6 male, 5 female)	45(NR)(NR)	22 (healthy elbows)
Struijs et al. (2005)[25], The Netherlands	57 (29 male, 28 female)	45.5(12.8)(NR)	57 (41 dominant, no bilateral affectation)	17(11.3)(NR) wks	NA	NA	57 (asymptomatic elbows)

Author, Year, Country	Number and gender of LE participants	Age of LE participants (mean, SD, range) in years	Number of symptomatic elbows	Duration of symptoms	Number and gender of healthy participants	Age of healthy participants (mean, SD, range) in years	Number of asymptomatic elbows
Levin et al. (2005)[32], United States	22 (10 male, 12 female)	46(NR)(30-59)	25 (3 with bilateral affectations)	Not indicated	10 (6 male, 4 female)	29.6(NR)(22-38)	32 (19 asymptomatic, 13 healthy elbows)
Miller et al. (2002)[33], United States	8 (5 male, 3 female)	46(NR)(38-63)	8 (no bilateral affectation)	7.6 mos. (NR)(3 wks-2 yrs), included those with medial epicondylitis	6 (3 males, 3 females)	29(NR)(25-30)	20 (8 asymptomatic, 12 healthy)
Connell et al. (2001)[30], Australia	76 (51 male, 25 female)	45.6(NR)(21-67)	72 (1 bilateral affectation)	7.1 mos(NR)(1 day to 9 yrs)	10 (6 male, 4 female)	36.4(NR)(NR)	10 (healthy)
Maffulli et al. (1990)[36], Italy	41 (39 male, 2 female)	24.3(7.3)(16-36)	41	2.2 mos(NR)(17 days-9.8 months)	NA	NA	N/A

Key: grp, group; mo(s), month(s); LE, lateral epicondylalgia; NA, not applicable; NR, not reported; SD, standard deviation; wk(s), week(s); yr(s), year(s); (+), positive; (-), negative; *, reported as median

Appendix 8. Sensitivity and Specificity of MSUS findings in elbows LE

Key:

Asx, asymptomatic
Calc, calcifications
CSA, cross sectional area
Ext, external
Int, internal
LE, Lateral Epicondylalgia
NA, not applicable
NR, not reported
NSx, non-symptomatic
SnS, sensitivity
SpC, specificity
* Patients with unilateral symptoms only were included for examinations of CEO thickness.
#non-symptomatic elbows of healthy participants
$ combined asymptomatic elbows of participants with LE and non-symptomatic elbows of healthy participants
^considered only those patients who received local corticosteroid injections
@asymptomatic elbows of participants with LE

Authors	N=Symptomatic elbows	SnS	N=Asx or NSx elbows	Spc
**MSUS techniques**				
**Gray-scale ultrasonography**				
Lee et al. (2010)[28]	51	0.76 (0.63-0.87)	63#	0.76 (0.64-0.86)
de Zordo et al. (2009)[16]	38	0.95 (0.89-0.98)	44$	0.89 (0.82-0.93)
du Toit et al. (2008)[31]	Variable*	0.81 (0.64-0.93)	Variable*$	0.63 (0.49-0.75)
Levin et al. (2005)[32] (first reading)	75	0.81 (0.71-0.89)	96$	0.46 (0.36-0.56)
Levin et al. (2005)[32] (second reading)	75	0.75 (0.63-0.84)	96$	0.42 (0.32-0.52)
Struijs et al. (2005)[25]	57	0.75 (0.62-0.86)	57@	0.81 (0.68-0.90)
**Gray scale ultrasonography + Power Doppler ultrasonography**				
Obradov and Anderson (2012)[26] (including hypoechoic regions)	49	1.00 (0.93-1.00)	10#	0.90 (0.55-1.00)
Obradov and Anderson (2012)[26] (excluding hypoechoic regions)	49	0.92 (0.81-0.97)	10#	0.90 (0.55-1.00)
Toprak et al. (2012)[27]	248	0.54 (0.48-0.60)	160#	0.88 (0.82-0.93)
du Toit et al. (2008)[31]	Variable*	0.97 (0.84-1.00)	Variable*$	0.61 (0.47-0.74)
**Real-time sonoelastography**				
de Zordo et al. (2009)[16]	38	1.00 (0.91-1.00)	44$	0.89 (0.75-0.96)
**MSUS findings**				
**Hypoechogenicity**				
Obradov and Anderson (2012)[26]	49	0.86 (0.73-0.94)	10#	1.00 (0.69-1.00)
Lee et al. (2011)[28]	51	0.35 (0.22-0.50)	63#	0.94 (0.85-0.98)
De Zordo et al. (2009)[16] (anterior section)	38	0.50 (0.33-0.67)	44$	1.00 (0.92-1.00)
De Zordo et al. (2009)[16] (middle section)	38	0.89 (0.75-0.97)	44$	0.89 (0.75-0.96)
De Zordo et al. (2009)[16] (posterior section)	38	0.53 (0.36-0.69)	44$	1.00 (0.92-1.00)
Noh et al. (2009)[18]	27	0.59 (0.39-0.78)	27#	0.85 (0.66-0.96)
Khoury and Cardinal (2009)[17]	8	1.00 (0.63-1.00)	8@	NR
Zeisig et al. (2006)[29]	22	1.00 (0.85-1.00)	22#	1.00 (0.85-1.00)

0

Authors	N=Symptomatic elbows	SnS	N=Asx or NSx elbows	Spc
**Neovascularity**				
du Toit et al. (2008)[31]	32	0.81 (0.64-0.93)	56	0.98 (0.90-1.00)
Zeisig et al. (2006)[28]	22	0.95 (0.77-1.00)	22[#]	0.91 (0.71-0.99)
Connell et al. (2001)[30]	72	0 (0.00-0.50)	10[#]	NR
**Thickness**				
Toprak et al. (2012)[27] (capitellar)	248	0.52 (0.46-0.58)	160[#]	0.80 (0.73-0.86)
Toprak et al. (2012)[27] (radiocapitellar)	248	0.38 (0.32-0.44)	160[#]	0.85 (0.79-0.90)
Lee et al. (2011)[28] (CSA cut-off)	51	0.86 (0.74-0.94)	63[#]	0.83 (0.71-0.91)
Lee et al. (2011)[28] (CSA max)	51	0.78 (0.65-0.89)	63[#]	0.95 (0.87-0.99)
Khoury and Cardinal (2009)[17]	8	1.00 (0.63-1.00)	8[@]	NR
du Toit et al. (2008)[31]	18	0.72 (0.47-0.90)	19	0.53 (0.29-0.76)
Miller et al. (2002)[33]	8	0.63 (0.24-0.91)	6[$]	1.00 (0.54-1.00)
Connell et al. (2001)[30]	72	0.35 (0.24-0.47)	10[#]	NR
Maffulli et al. (1990)[36] (enthesopathy)	41	0.12 (0.40-0.26)	0	NA
Maffulli et al. (1990)[36] (tendonitis)	41	0.37 (0.22-0.53)	0	NA
Maffulli et al. (1990)[36] (peritonitis)	41	0.10 (0.03-0.23)	0	NA
**Enthesopathy**				
Khoury and Cardinal (2009)[17]	8	0.63 (0.24-0.91)	8[@]	NR
Noh et al. (2009)[18]	27	0.56 (0.35-0.75)	27[#]	0.85 (0.66-0.96)
Tarhan et al. (2009)[35]	52	0.08 (0.02-0.19)	0	0

Authors	N=Symptomatic elbows	SnS	N=Asx or NSx elbows	Spc
**Cortical irregularities**				
Obradov and Anderson (2012)[26]	49	0.18 (0.09-0.32)	10#	1.00 (0.69-1.00)
Lee et al. (2011)[28]	51	0.18 (0.08-0.31)	63#	0.95 (0.87-0.99)
Connell et al. (2001)[30]	72	0.22 (0.13-0.34)	10#	NR
**Cortical irregularities or bony spurs**				
Toprak et al. (2012)[27]	248	0.55 (0.48-0.61)	160#	0.91 (0.86-0.95)
du Toit et al. (2008)[31]	32	0.63 (0.44-0.79)	56$	0.63 (0.49-0.75)
**Cortical spurs**				
Zeisig et al. (2010)[34]	28	0.08 (0.03-0.17)	0	NA
Connell et al. (2001)[30]	72	0.25 (0.11-0.45)	10#	NR
**Tear**				
Obradov and Anderson (2012)[26]	49	0.14 (0.06-0.27)	10#	1.00 (0.69-1.00)
Toprak et al. (2012)[27]	248	0.35 (0.29-0.41)	160#	1.00 (0.98-1.00)
**Partial tear**				
Khoury and Cardinal (2009)[17]	8	0.38 (0.09-0.76)	8@	NR
Tarhan et al. (2009)[35]	52	0.10 (0.03-0.21)	0	NA
Connell et al. (2001)[30]	72	0.25 (0.16-0.37)	10#	NR
**Full thickness tear**				
Tarhan et al. (2009)[35]	52	0.00 (0.00-0.70)	0	NA
Connell et al. (2001)[30]	72	0.03 (0.00-0.10)	0	NR
**Adjacent fluid**				
Lee et al 2010[28]	51	0.04 (0.01-0.13)	63	1.00 (0.94-1.00)

Key: NA, not applicable; NR, not reported

Appendix 9. Criteria used to determine abnormal MSUS findings

Hypoechogenicity		
Authors	*Mark*	*Description*
Noh et al., 2009	A	focal hypoechogenicity on normal background
Connell et al., 2001	A	focal hypoechogenicity on normal background
Struijs et al., 2005	A	focal hypoechogenicity on normal background
De Zordo et al., 2009 (anterior section)	B	focal lesions with areas of degeneration and partial rupture
De Zordo et al., 2009 (middle section)	B	focal lesions with areas of degeneration and partial rupture
De Zordo et al., 2009 (posterior section)	B	focal lesions with areas of degeneration and partial rupture
Lee et al., 2011	X	as a rounded spot and not associated with disruption
Khoury and Cardinal 2009	X	hypoechogenicity with or without tendon thickening
Miller et al., 2002 (deep to tendon)	X	hypoechoic fluid between the tendon and the epicondyle
Miller et al., 2002 (general hypoechogenicity)	X	relative to the contralateral elbow
Zeisig et al., 2006	X	with diffuse heterogeneity and hypoechogenicity
Obradov and Anderson 2012	X	focal lesion
**Calcifications**		
Authors	*Mark*	*Description*
Lee et al., 2011	A	intratendinous
Khoury and Cardinal 2009	A	intratendinous
Obradov and Anderson 2012 (int calc)	A	intratendinous
Toprak et al., 2012	A	intratendinous
Connell et al., 2001	X	focal areas
Tarhan et al., 2009	X	focal areas
Struijs et al., 2005	X	no description
Zeisig et al., 2006	X	with local steriod injections only
Obradov and Anderson 2012	X	no description
Obradov and Anderson 2012 (ext calc)	X	external

Neovascularity		
Authors	*Mark*	*Description*
Khoury and Cardinal 2009	X	no description
du Toit et al., 2008	A	presence of neovessel diameter of more than 1 mm
Zeisig et al., 2010	X	high blood flow
Zeisig et al., 2006	X	inside and outside the dorsal part of the tendon
Obradov and Anderson 2012	X	no description
Obradov and Anderson 2012 (including HE regions)	X	including hypoechoic regions
Obradov and Anderson 2012 (excluding HE regions)	X	excluding hypoechoic regions
Toprak et al., 2012	A	new vessel formation thicker than 1mm within or near the CEO
Connell et al., 2001	X	no description
**Thickness**		
Authors	*Mark*	*Description*
Connell et al., 2001	A	Enlarged or attenuated if there was a 10% difference with normal elbow
du Toit et al., 2008	A	Enlarged or attenuated if there was a 10% difference with normal elbow
Lee et al., 2011 (CSA cut-off)	X	quantitative, transverse images
Lee et al., 2011 (CSA max)	X	quantitative, transverse images
Toprak et al., 2012 (capitellar)	X	quantitative, longitudinal images
Toprak et al., 2012 (radiocapitellar)	X	quantitative, longitudinal images
Khoury and Cardinal 2009	X	measured with compression
Miller et al., 2002	X	thickening or thinning
Maffulli et al., 1990 (enthesopathy)	X	proximal part of the tendon was enlarged
Maffulli et al., 1990 (tendonitis)	X	tendon of ECRB was enlarged
Maffulli et al., 1990 (peritonitis)	X	peritendinous lining was enlarged

Enthesopathy		
Authors	*Mark*	*Description*
Struijs et al., 2005	A	proximal part of the tendon enlarged with alterations in echogenicity
Tarhan et al., 2009	A	proximal part of the tendon enlarged with alterations in echogenicity
Maffulli et al., 1990	A	proximal part of the tendon enlarged with alterations in echogenicity
Khoury and Cardinal 2009	X	no description
Noh et al., 2009	X	Echogenicity at the ECRB insertion
**Cortical irregularities**		
Authors	*Mark*	*Description*
Lee et al., 2011	A	adjacent bone irregularity
Connell et al., 2001	A	irregular bony surface
Obradov and Anderson 2012	A	cortical bone irregularity
**Cortical spurs**		
Authors	*Mark*	*Description*
Connell et al., 2001	A	spur formation
Zeisig et al., 2010	A	spur formation
**Bone changes (cortical irregularities or spurs)**		
Authors	*Mark*	*Description*
Toprak et al., 2012	A	any irregularity of the bony surface
du Toit et al., 2008	A	cortical spurring or any bone irregularity
**Tear**		
Authors	*Mark*	*Description*
Obradov and Anderson 2012	X	no description
Toprak et al., 2012	X	loss of fibrillar continuity, if within the tendon, intrasubstantial
**Partial tear**		
Authors	Mark	Description
Khoury and Cardinal 2009	X	tear
Connell et al., 2001	A	focal anechoic area with no fibers intact or an echogenic irregular band that could run either horizontally or longitudinally in the CEO
Tarhan et al., 2009	A	focal anechoic area with no fibers intact or an echogenic irregular band that could run either horizontally or longitudinally in the CEO
**Full thickness tear**		
Authors	*Mark*	*Description*
Connell et al., 2001	A	Distinct complete interval traversing or extending through the full width of the CEO. Confirmation was performed in at least two planes of imaging.
Tarhan et al., 2009	A	Distinct complete interval traversing or extending through the full width of the CEO. Confirmation was performed in at least two planes of imaging.

Note: A and B mark those diagnostic criteria which were similar. X marks those diagnostic criteria which were different from each other and thus, cannot be pooled into one classification. *Key:* CEO, common extensor origin; ECRB, Extensor Carpi Radialis Brevis; MSUS, musculoskeletal

Appendix 10. Similarities of collected MSUS data in 15 diagnostic studies

	MSUS Findings								Other variables obtained during data collection					
	HE	Calc	Neov	Enth	Thick	Cortical irreg	Spur	Tear	Inclusion Criteria reported (Y/N)	Reference standard used	Frequency of linear transducer head reported (Y/N)	Qualified? Y, N	Age (if mean is 35-60 years old)	Duration of symptoms (Y if more than or equal to 6 weeks)
Obradov and Anderson (2012)	X	A	X	--	--	A	--	X	Y	B	Y	Y	Y	Y
Toprak et al. (2012)	--	A	A	--	B	--	--	X	Y	A	Y	Y	Y	Y
Lee et al. (2011)	X	A	--	--	B	A	--	--	Y	A	Y	Y	Y	Y
Zeisig et al. (2010)	--	--	X	--	--	--	A	--	Y	C	Y	Y	Y	Y
Noh et al. (2009)	A	--	--	X	--	--	--	--	Y	B	N	Y	Y	Y
De Zordo et al. (2009)	B^ (anterior, middle, posterior sections)	--	--	--	--	--	--	--	Y^	A^	Y^	Y^	Y^	Y^
Khoury and Cardinal (2009)	X	A	X	X	X	--	--	X	Y	A	Y	Y	Y	--
Tarhan et al. (2009)	--	X	--	A	--	--	--	A,B	Y	A	Y	Y	Y	Y
du Toit et al. (2008)	--	--	A	--	A	--	--	--	Y	A	Y	Y	Y	Y
Zeisig et al. (2006)	X	X	X	--	--	--	--	--	Y	A	Y	Y	Y	Y
Struijs et al. (2005)	A	X	--	A	--	--	--	--	Y	A	Y	Y	Y	Y
Levin et al. (2005)	--	--	--	--	--	--	--	--	Y	A	Y	Y	Y	--
Miller et al. (2002)	X	--	--	--	X	--	--	--	Y	A	Y	Y	Y	Y
Connell et al. (2001)	A	X	X	--	A	A	A	A,B	Y	A	Y	Y	Y	Y
Maffulli et al. (1990)	--	--	--	A	X	--	--	--	Y	A	Y	Y	N	Y

Note: A and B marks report studies which used similar diagnostic criteria. X mark indicates those studies which used different diagnostic criteria and thus not pooled. *Key*: Calc, calcifications; Enth, enthesopathy; HE, hypoechogenicity; irreg, irregularities; MSUS, musculoskeletal ultrasound; N, no; Neov, neovascularity; Y, Yes;--, not reported; ^, used real time sonoelastography

Appendix 11. Forest Plots of on Diagnostic Validity of Abnormal MSUS findings

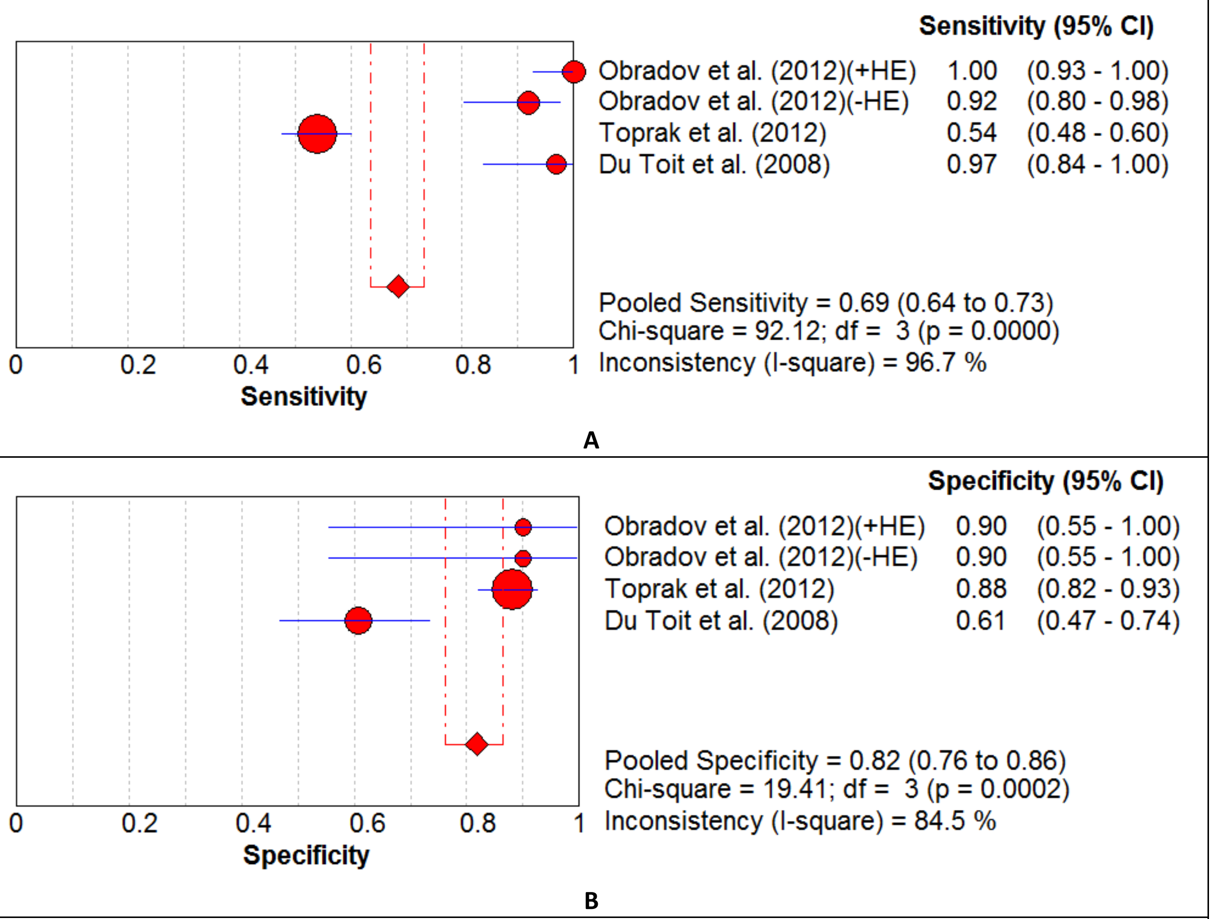

Figure 3. Forest plots for combined grayscale changes and Power Doppler Ultrasonography including a study which used sonographic probe induced tenderness. A. Sensitivity, B. Specificity
Key: HE, hypoechogenicity; (+), positive; (-), negative

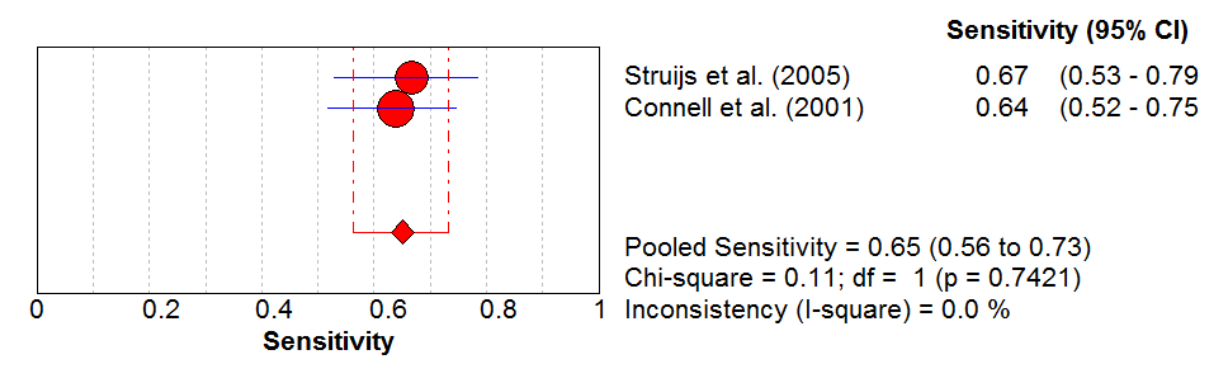

Figure 4. Forest plot for pooled sensitivity of CEO's hypoechogenicity described in the studies as focal lesion on normal background.

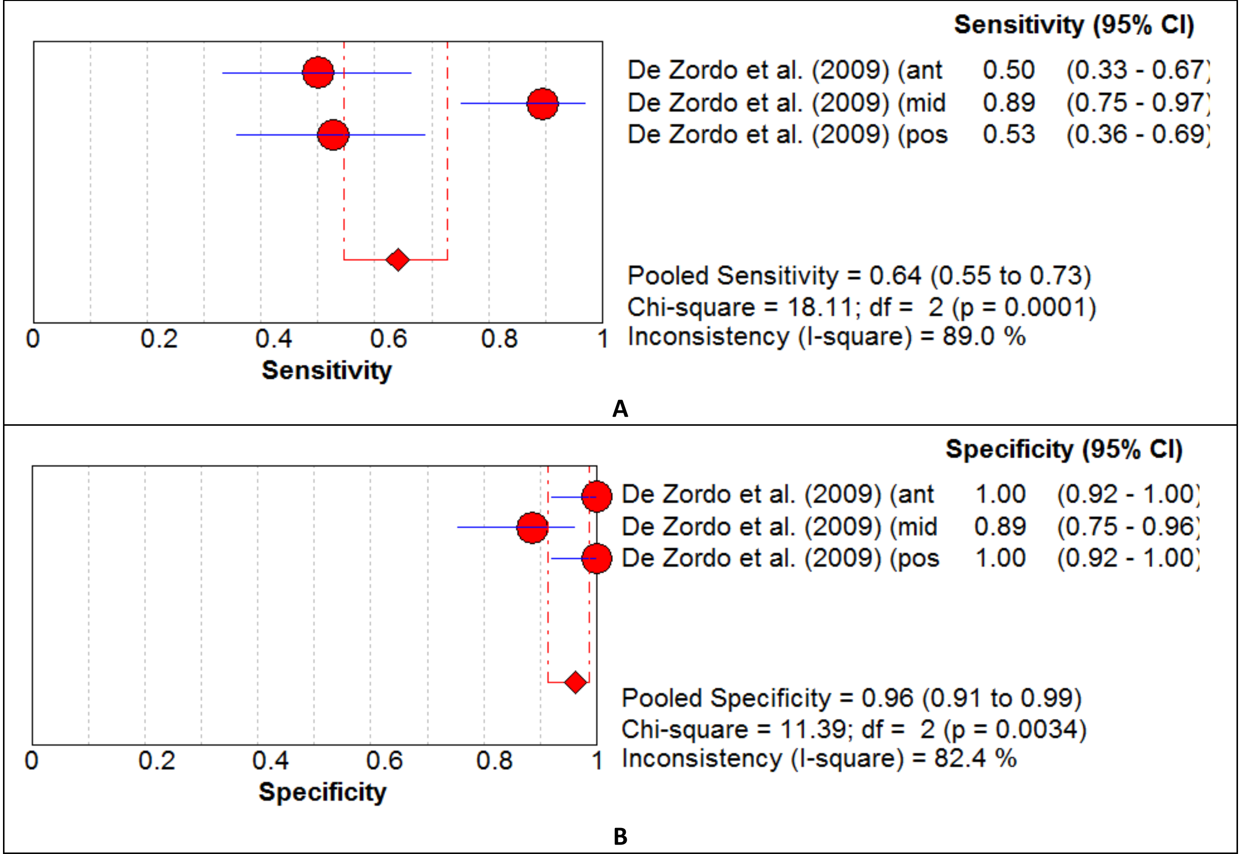

Figure 5. Forest plots for CEO hypoechogenicity described as focal lesion in areas of degeneration and partial rupture. A. Sensitivity, B. Specificity
Key: ant, anterior; mid, middle; pos, posterior

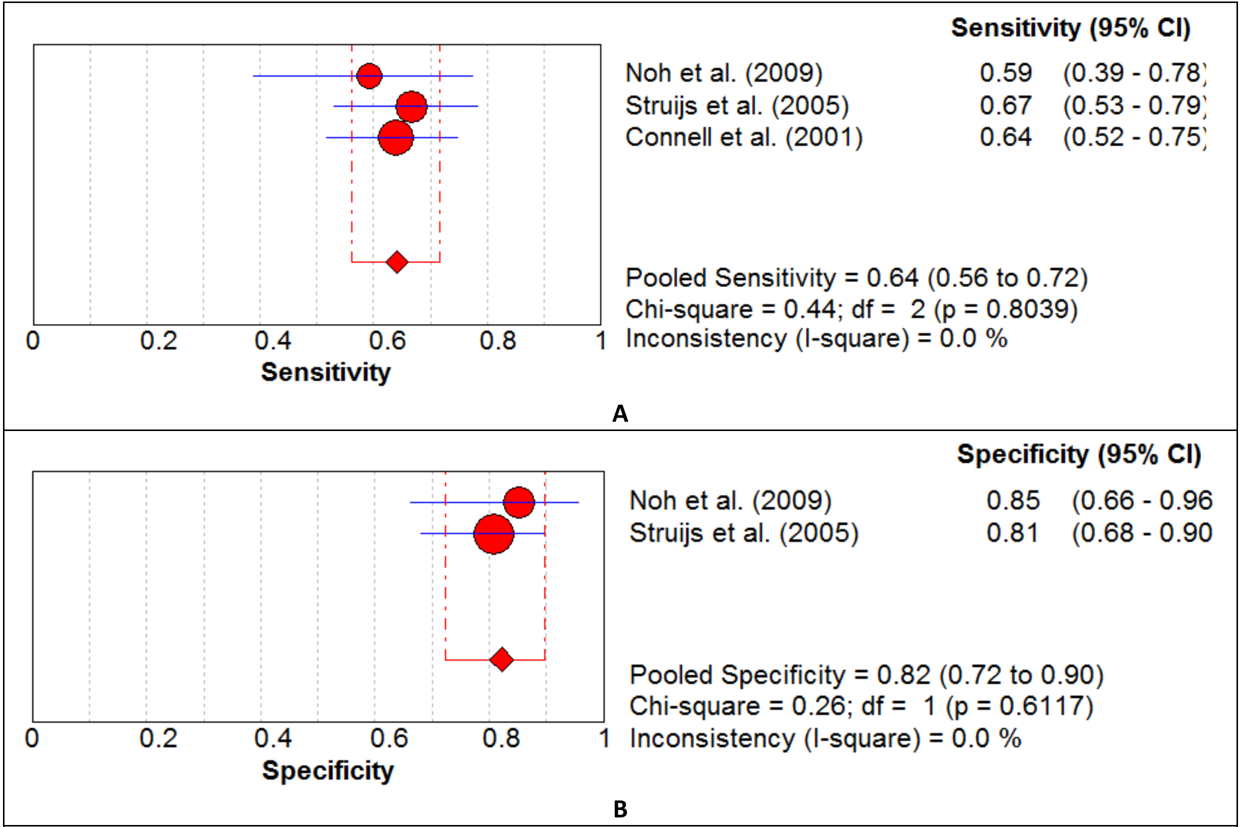

Figure 6. Forest plots for CEO hypoechogenicity described in the studies as focal lesion on normal background where data from a study which used sonographic probe induced tenderness was combined in the pooled analysis. A. Sensitivity, B. Specificity

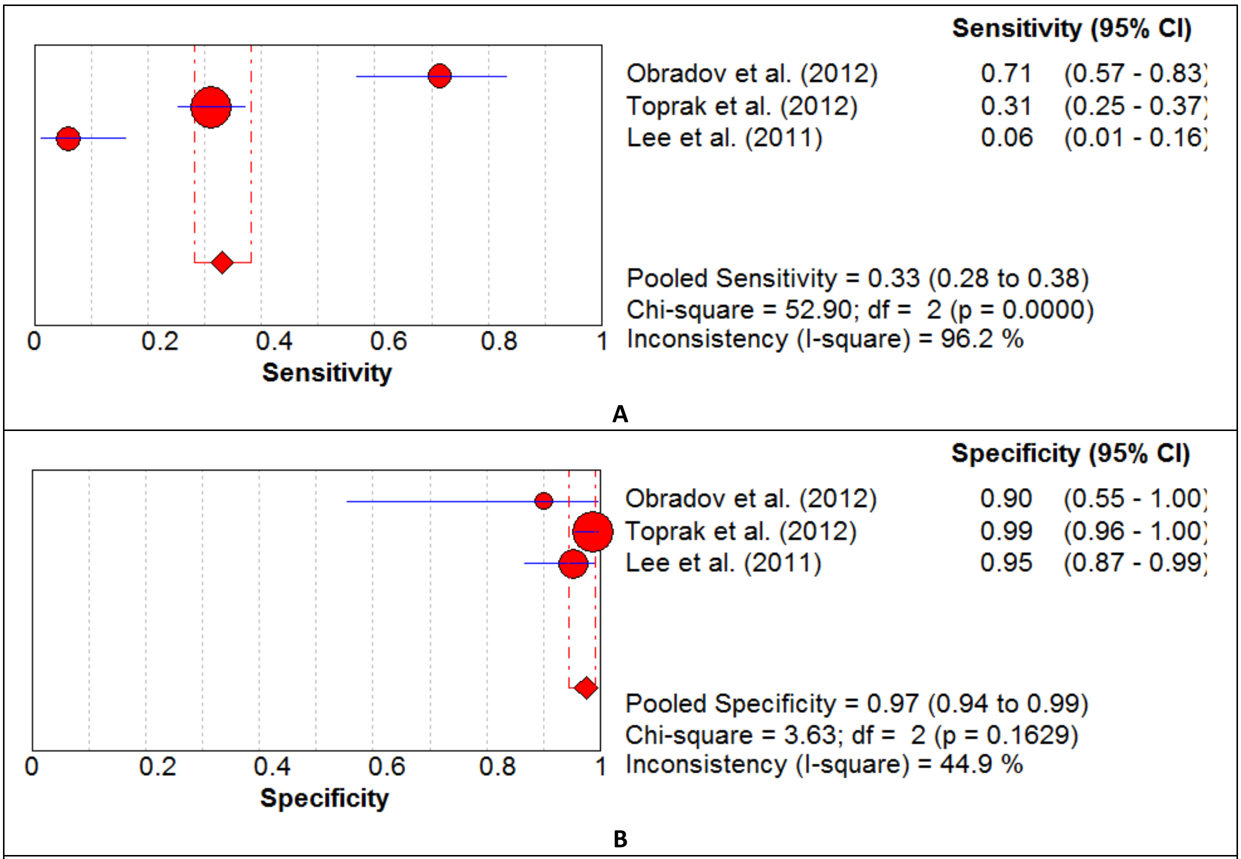

Figure 7. Forest plots for CEO calcification. A. Sensitivity, B. Specificity

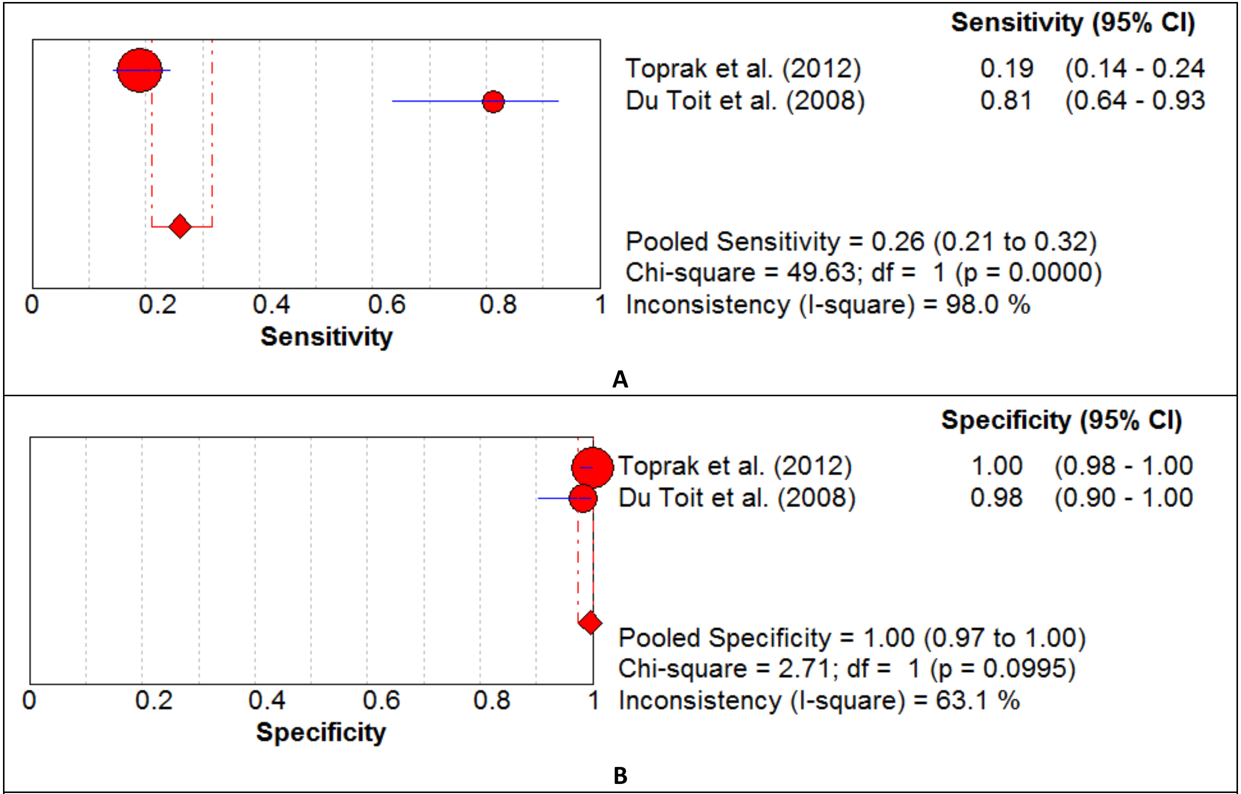

Figure 8. Forest plot for CEO neovascularity. A. Sensitivity, B. Specificity

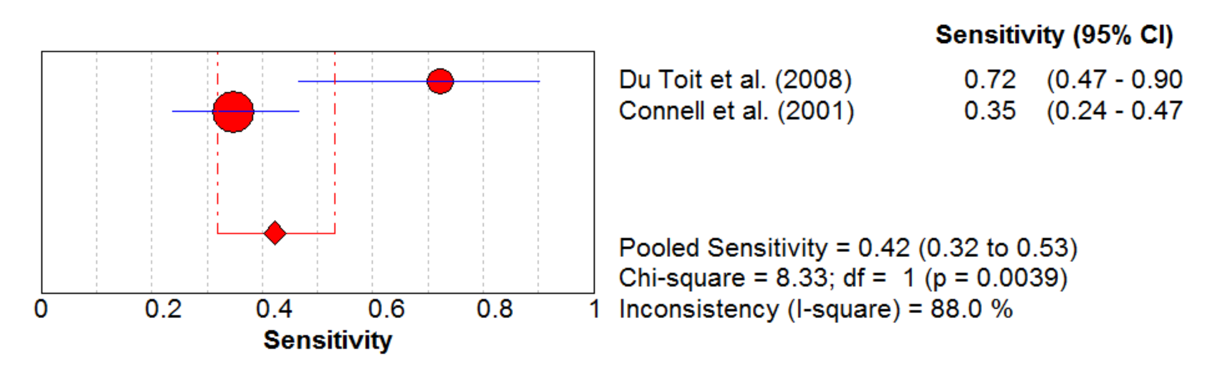

Figure 9. Forest plot on pooled sensitivity of CEO thickness based on qualitative observation.

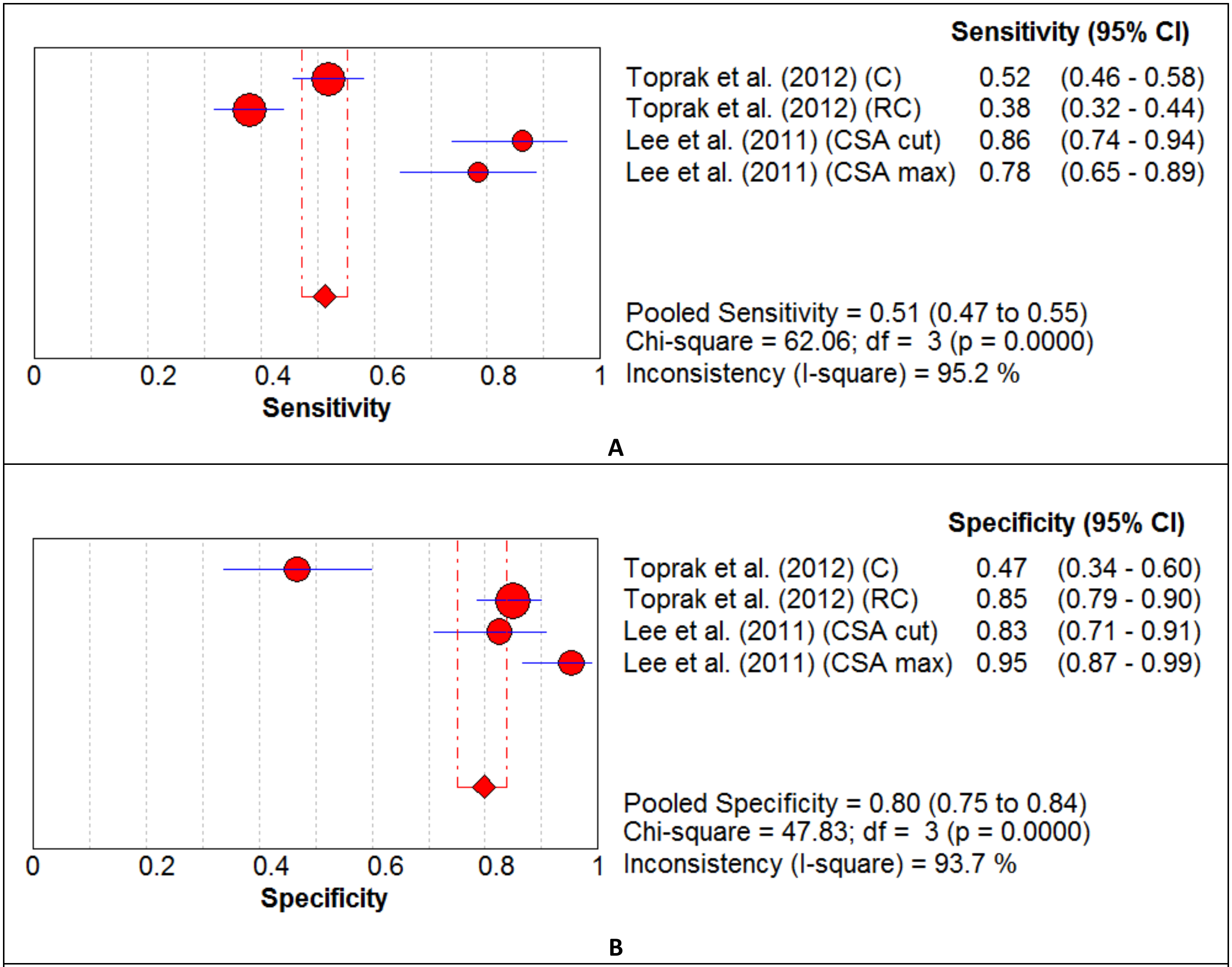

Figure 10. Forest plots of CEO thickness based on quantitative measurements. A. Sensitivity, B. Specificity. *Key:* C, capitellar; CSA, cross-sectional area; cut, cut-off; RC, radiocapitellar

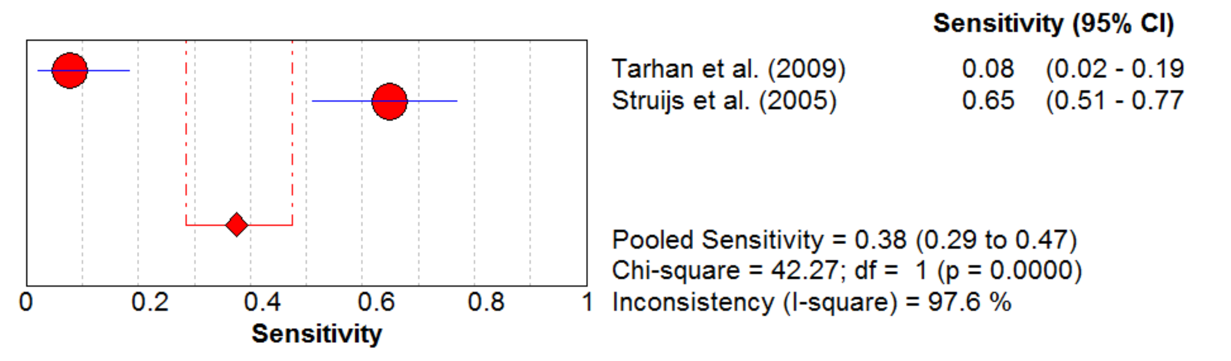

Figure 11. Forest plot for pooled sensitivity of CEO enthesopathy

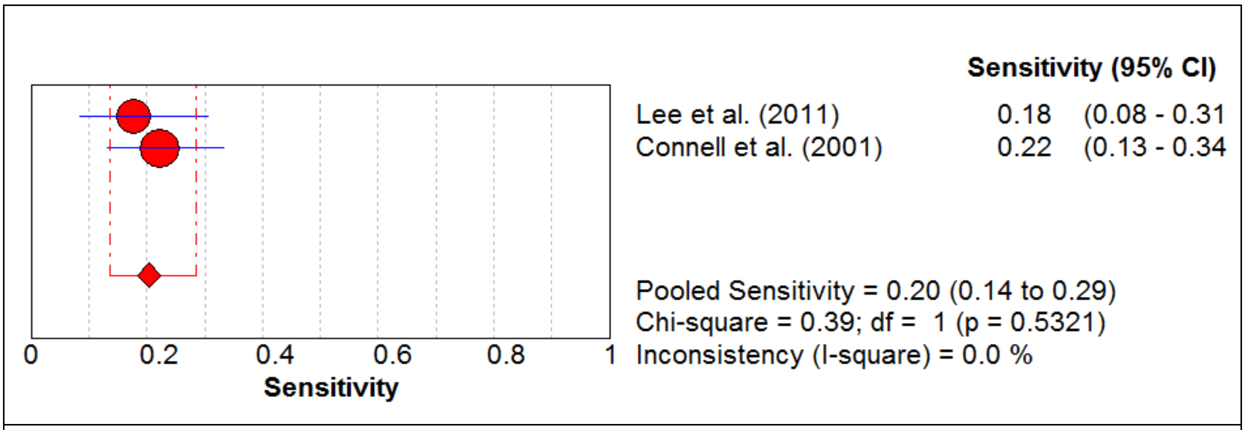

Figure 12. Forest plot for pooled sensitivity of cortical irregularities.

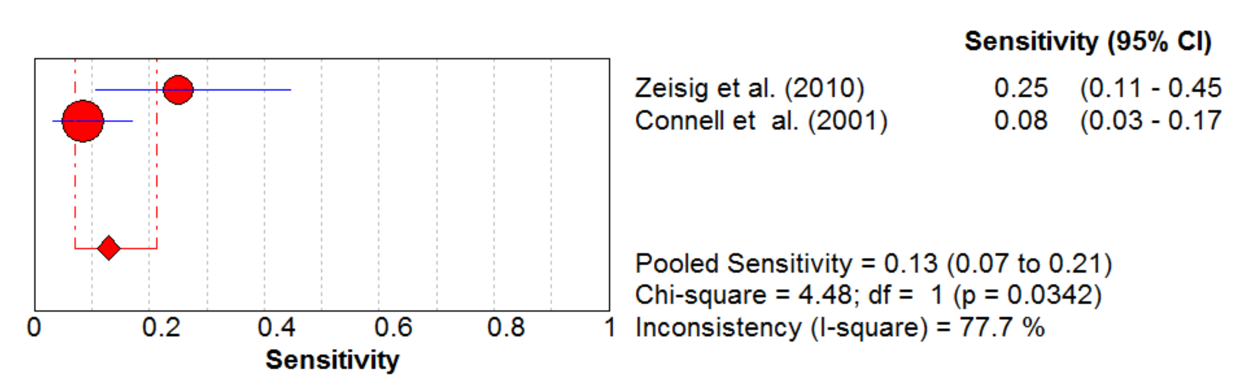

Figure 13. Forest plot for pooled sensitivity of cortical spurs.

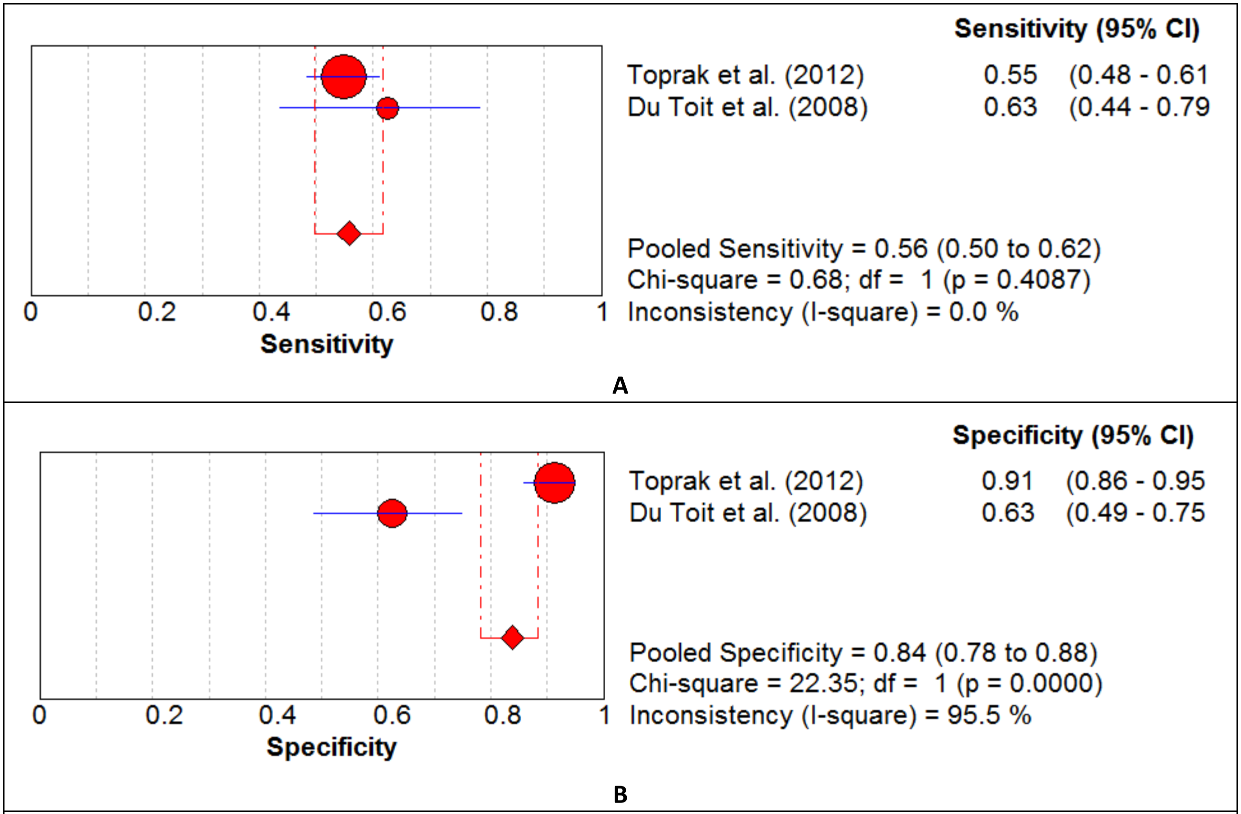

Figure 14. Forest plots for presence of combined cortical irregularities and cortical spurs. A. Sensitivity, B. Specificity

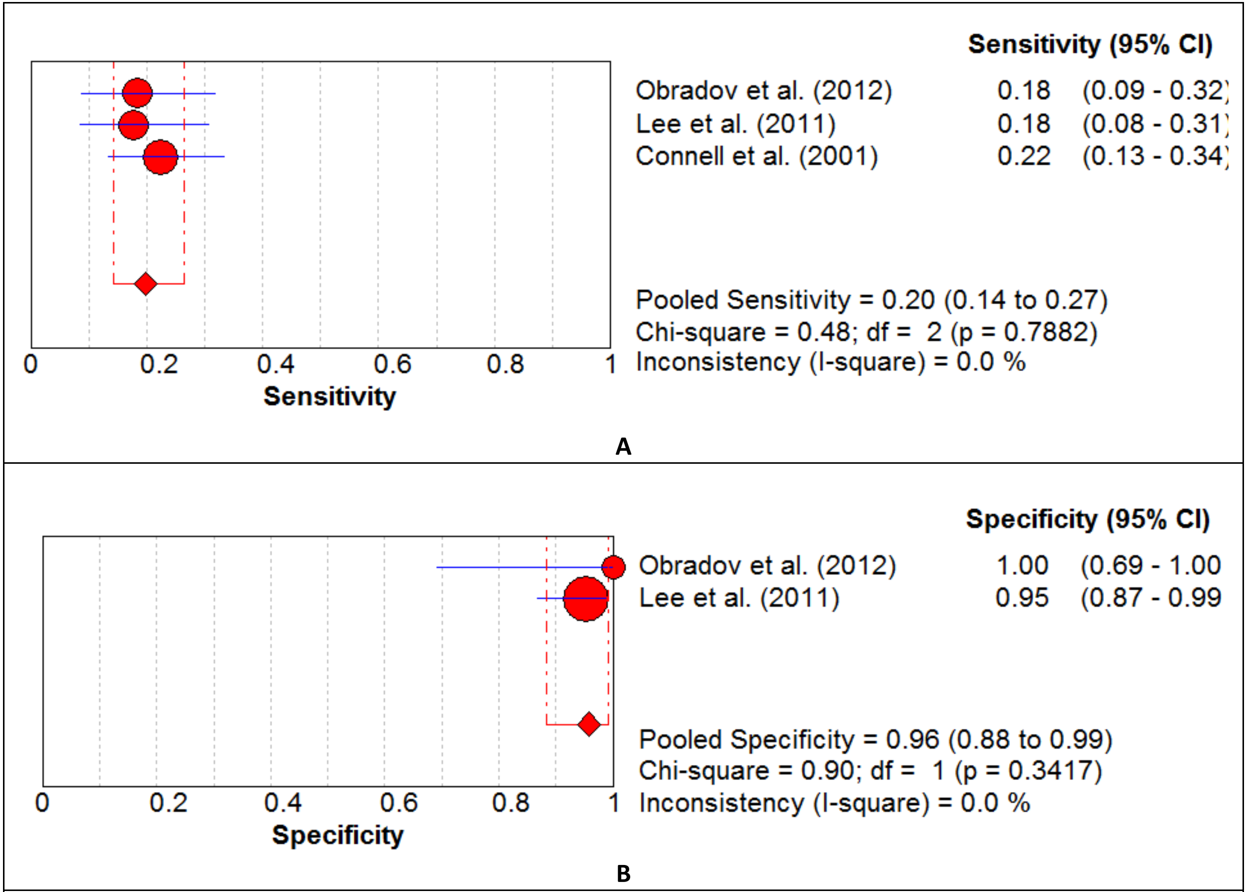

Figure 15. Forest plots for cortical irregularities including a study which incorporated sonographic probe tenderness in the reference standard. A. Sensitivity, B. Specificity

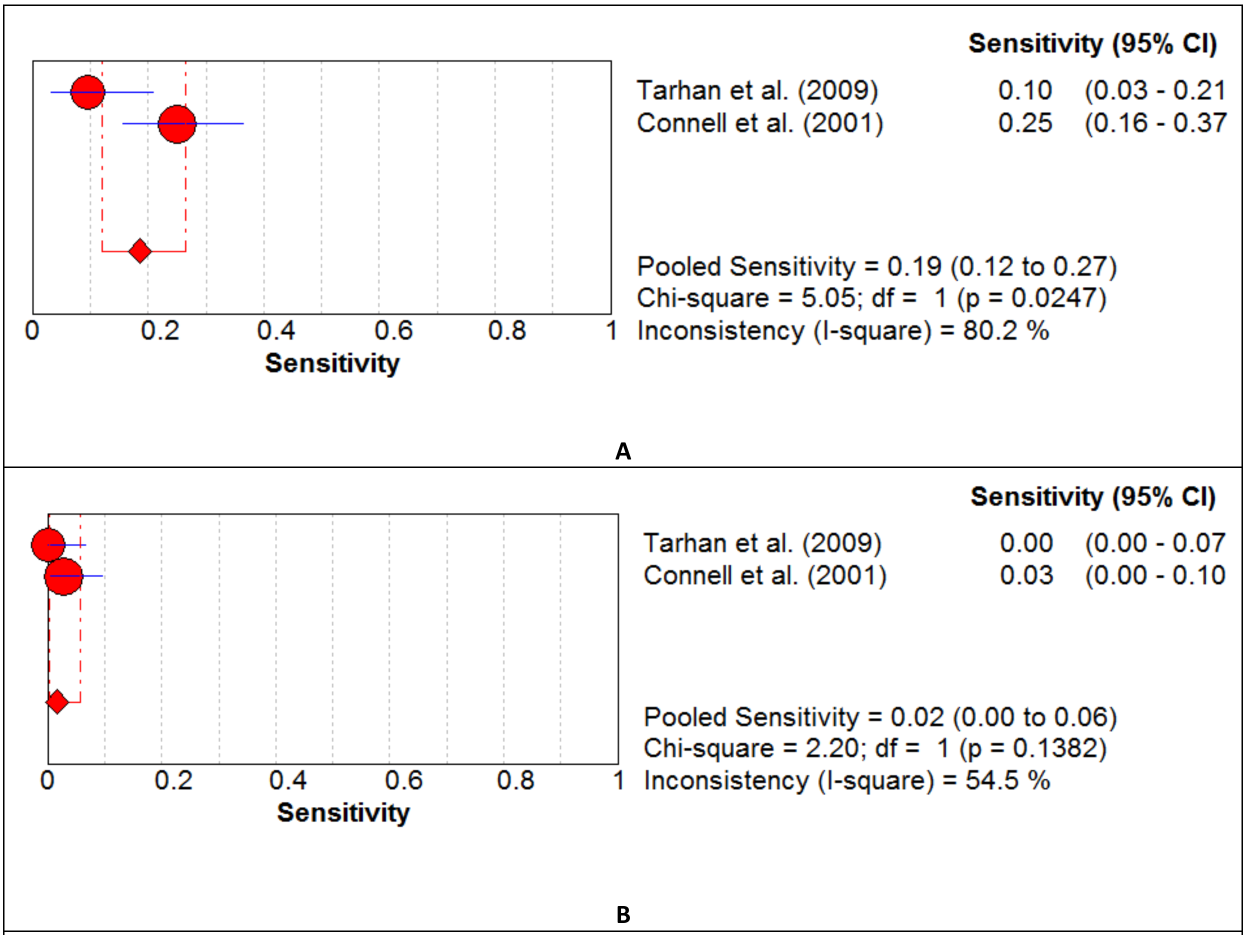

Figure 16. Forest plots for pooled sensitivity of CEO tear. A. Partial tear B. Full tear
